# To reveal biomarkers related to macrophage and lactic acid metabolism in renal fibrosis and explore their mechanisms

**DOI:** 10.3389/fimmu.2025.1609903

**Published:** 2025-07-18

**Authors:** Chen Yong, Yongfei Yu, Yuan Wei, Guoshun Huang, Lianghui Shu, Kun Gao, Enchao Zhou

**Affiliations:** ^1^ Division of Nephrology, Affiliated Hospital of Nanjing University of Chinese Medicine, Jiangsu Province Hospital of Chinese Medicine, Nanjing, China; ^2^ Jiangsu University Key Laboratory of Tonifying Kidney and Anti-senescence, Nanjing University of Chinese Medicine, Nanjing, China; ^3^ The First School of Clinical Medicine, Nanjing University of Chinese Medicine, Nanjing, China; ^4^ Department of Nephrology, Wuxi Ninth People’s Hospital Affiliated of Soochow University, Wuxi, China; ^5^ Inheritance Studio of Traditional Chinese Medicine Master Yanqin Zou, Jiangsu Province Hospital of Chinese Medicine, Nanjing, China

**Keywords:** lactate metabolism, macrophages, biomarkers, machine learning, renal fibrosis

## Abstract

**Introduction:**

Lactate can influence the fibrotic process by regulating cellular metabolism, inflammatory responses, and cell proliferation, which may be closely related to macrophage function in diseases. Therefore, this research sought to identify biomarkers linked to lactate metabolism and macrophages in renal fibrosis (RF).

**Methods:**

Firstly, key modular genes associated with macrophage score and lactate metabolism score were identified by combining single-sample gene set enrichment analysis (ssGSEA) and weighted gene co-expression network analysis. Then, candidate genes were obtained by overlapping them with differentially expressed genes between RF and control groups. Subsequently, candidate genes were incorporated into machine learning algorithms to identify key feature genes associated with RF. Expression analysis was then completed to determine biomarkers for this study. Furthermore, the relationship between biomarkers and RF was elucidated by a series of bioinformatics methods, including enrichment analysis, immunosignature analysis, and molecular regulatory analysis. Finally, we validated these key biomarkers in animal experiments.

**Results:**

The ssGSEA results showed significantly higher macrophage score and lower lactate metabolism score in the RF samples compared to control samples. Next, AGR3, CD74, and SYT11 were identified as biomarkers for this study because they had consistent expression trends in GSE76882 and GSE135327 datasets and were significantly different between RF and control samples. Moreover, receiver operating characteristic curves showed their excellent accuracy in predicting the occurrence of RF. Subsequent enrichment analysis revealed that three biomarkers were collectively enriched to 50 signaling pathways, including “Toll-like receptor signaling pathway”, “oxidative phosphorylation”, and “P53 signaling pathway”. Notably, CD74 showed a significant positive correlation with macrophages. In lncRNA-miRNA-mRNA network, multiple relationship pairs could be found, e.g., hsa-miR-548x-3p and hsa-miR-548aj-3p were regulators of AGR3, as well as multiple lncRNAs (PCAT6, POLR2J4, SMIM25) could co-regulate CD74 through hsa-miR-4731-5p. Animal experiments also confirmed that the expression of key biomarkers were significantly elevated in the RF rat/mice model. Moreover, the localization and expression of these biomarkers were related to infiltrating inflammatory cells in the kidney tissue.

**Conclusion:**

In this study, we found that AGR3, CD74, and SYT11 were biomarkers associated with lactate metabolism and macrophages in RF, providing valuable insights for further RF research.

## 1 Introduction

Chronic kidney disease (CKD) is a chronic (more than 3 months) disorder of kidney structure and function due to various causes. The global prevalence rate of CKD is more than 10%, especially in the elderly, hypertensive and diabetic populations, which poses a serious threat to human health (1). Renal fibrosis (RF) is a common pathological change in vaeious progressive forms of CKD, manifested by destruction of normal renal tissue, fibroblast proliferation and excessive deposition of extracellular matrix (ECM) (2). Extensive renal fibrosis is the core pathological mechanism for the continuous deterioration of renal function and even progression to end-stage renal disease in patients with CKD (3). There is currently a lack of effective drugs for the treatment of RF. Although several clinical trials have been conducted to evaluate targeted treatment strategies for fibrosis drivers and signaling pathways, their efficacy and safety have been unsatisfactory (4, 5). The pathogenesis of RF involves multiple aspects, but not limited to, excessive EMC deposition, activation and proliferation of renal innate cells, and a persistent inflammatory response. However, the exact pathological mechanism of RF is not fully understood. Therefore, there is an urgent need to further explore the mechanisms underlying the development and progression of RF so that more effective treatment strategies can be developed to improve the quality of life and prognosis of patients with CKD.

Macrophages are phagocyte subsets of white blood cell. They are immune cells that are widely distributed in all tissues and organs of the body. Their main functions include phagocytosis and removal of pathogens, damaged cells and cell debris, while also secreting a variety of cytokines (such as TNF-α, IL-6) that regulate immune and inflammatory responses in various tissues (6–8). Macrophages play a key role in maintaining tissue homeostasis and repair processes (9, 10). There is a close relationship between macrophages and RF. Studies have shown that macrophages can promote the damage of renal tubular epithelial cells (RTECs) and the activation of interstitial fibroblasts by secreting pro-inflammatory cytokines and pro-fibrotic factors in pathological conditions, thus accelerating the process of RF (11). In addition, macrophages can further exacerbate RF by affecting the deposition and degradation of ECM (12). Lactic acid metabolism refers to the process in which cells produce lactic acid through glycolysis under hypoxic or aerobic conditions (13). Lactic acid is not only a product of cellular energy metabolism, but also plays an important role in a variety of physiological and pathological processes (14, 15). Studies have found that lactic acid can promote the proliferation of fibroblasts and collagen synthesis by activating specific signaling pathways, thus exacerbating RF (16). In addition, lactic acid may also be indirectly involved in the pathological process of RF by affecting the function of immune cells, such as regulation of macrophage polarisation, induction of metabolic reprogramming of T cells, inhibition of dendritic cell maturation, etc. (17). Lactic acid metabolism plays an important role in the physiological and pathological processes of macrophages. Macrophages produce large amounts of lactic acid during phagocytosis and metabolism, which in turn can affect macrophage function and phenotype (18). For example, high levels of lactic acid can promote the polarization of macrophages towards pro-inflammatory phenotypes, thereby exacerbating inflammatory responses and tissue damage (19). Overall, the link between lactate metabolism and macrophages provides a new perspective for our understanding of RF. In-depth study of the specific mechanisms and interactions between them will help to provide new ideas and methods for the treatment of RF.

In this study, biomarkers associated with macrophage and lactate metabolism were identified through a series of bioinformatic approaches using data related to RF in public databases. On this basis, the molecular mechanisms of the biomarkers were investigated by enrichment analysis, immune infiltration analysis, regulatory network analysis, etc. Finally, the relevant biomarkers were verified by animal experiments. The results are expected to provide a new reference for the clinical diagnosis and treatment of RF.

## 2 Materials and methods

### 2.1 Data collection

The RF-related datasets GSE76882 and GSE135327 were downloaded from the gene expression omnibus (GEO, http://www.ncbi.nlm.nih.gov/geo/) database using the GEOquery package (v 3.21) (20). Specifically, the GSE76882 dataset (downloaded on June 12, 2024), based on the GPL13158 platform, contained 274 samples. A total of 42 renal tissue biopsy samples with interstitial fibrosis/tubular atrophy (recorded as RF samples) and 99 normal samples were selected for analysis. The GSE135327 dataset (downloaded on July 24, 2024) (platforms: GPL11154 and GPL21290) included 30 samples, among which 18 were interstitial fibrosis samples (recorded as RF samples) and 12 were normal renal tissue biopsy samples. The platform files were then downloaded, and gene Symbol and probe ID information was extracted. Probe IDs were converted to gene Symbols, and duplicate genes were removed by retaining the maximum value for each gene. Subsequently, sample information and grouping information were extracted for subsequent analysis. In molecular signatures database (MsigDB, http://software.broadinstitute.org/gsea/msigdb), we searched “lactate” and selected the genes contained in five pathways: LACTATE_METABOLIC_PROCESS, HP_INCREASED_SERUM_LACTATE, HP_ LACTIC_ACIDOSIS, HP_LACTICACIDURIA, and HP_SEVERE_LACTIC_ACIDOSIS, followed by collecting 320 lactate metabolism-related genes (LMRGs) by removing duplicates (**Supplementary Table S1**).

### 2.2 Single-sample gene set enrichment analysis

In order to obtain most significant differential immune cells, ssGSEA algorithm was employed via GSVA package (v 1.42.0) to calculate the scores of the infiltration levels of 28 immune cells in all samples for GSE76882 dataset (21, 22). The most significant differential immune cells were then obtained by comparing the differences in immune cells between RF and control samples using the Wilcoxon test (*P* < 0.05). Additionally, LMRGs with significant differences between RF and control samples were analyzed by the Wilcoxon test (*P* < 0.05). The ssGSEA enrichment scores of these significantly differentially expressed LMRGs were calculated using the GSVA package (v 1.42.0) to obtain LMRGs scores (21). Then, the differences in LMRGs scores between RF and control samples were analyzed by the Wilcoxon test (*P* < 0.05).

### 2.3 Weighted gene co-expression network analysis

To identify genes associated with both most significant differential immune cells and LMRGs scores, WGCNA was performed using the differential immune cells and LMRGs scores as traits via the WGCNA package (v 1.7.1) in GSE76882 (23). Firstly, cluster analysis was performed on all samples to check for and eliminate outliers, aiming to ensure accuracy in downstream analysis. Next, the relationship between the soft-thresholding power (β), scale-free network evaluation coefficient R², and mean connectivity was established. The optimal soft thresholding was obtained when the scale-free network evaluation coefficient R² was set to 0.85, the critical value at which R² first exceeded 0.85, and the mean connectivity of the co-expression network approached zero. Subsequently, based on the optimal soft threshold, hierarchical clustering algorithms were applied to perform cluster analysis on genes. With parameters set as minModuleSize=50, deepSplit=2, and mergeCutHeight=0.15, genes with similar expression patterns were grouped into the same modules, which were labeled with different colors. A correlation heatmap between traits and modules was constructed, and then modules with the most significant correlations with traits were further screened out as key modules (|correlation (cor)| > 0.65, *P* < 0.05). Genes in key modules were recorded as key module genes for subsequent analysis.

### 2.4 Differential expression analysis

To obtain the differential expression analysis between the RF and control groups, on the basis of the gene expression matrix in GSE76882, differentially expressed genes (DEGs) between RF and control groups were mined applying limma package (v 3.54.0) (24), with screening cutoffs of adj.*P* value < 0.05 and |log_2_FoldChange(FC)| > 0.5. To understand the distribution of DEGs from a holistic perspective, volcano plot and heat map of DEGs were generated by ggplot2 package (v 3.4.1) and pheatmap package (v 1.0.12), respectively (25, 26). The top 10 upregulated genes and 4 downregulated genes ranked by log_2_FC were annotated on the volcano plot. Next, the Venn diagram created by the VennDiagram package (v 1.7.1) displayed the intersecting genes of key module genes and DEGs, which were recorded as candidate genes (27).

### 2.5 Functional enrichment and protein-protein interactions analyses

To investigate the functions and pathways involved in candidate genes, we performed Gene Ontology (GO) and Kyoto Encyclopedia of Genes and Genomes (KEGG) enrichment analyses via the clusterProfiler package (v 4.2.2) (28), with screening criteria set at *P* < 0.05. GO involved three components: biological process (BP), cellular component (CC), and molecular function (MF). The top 5 results of each part of GO were displayed in ascending order of *P*-values. Following this, a PPI network was created through imputing candidate genes into STRING online site (http://www/string-db.org/), with the aim of exploring candidate gene interactions at the protein level (Species: Homosapiens, confidence level ≥ 0.4). Cytoscape software (v 3.5.2) was employed to accomplish the visualization of PPI network (29).

### 2.6 Machine learning algorithms

Machine learning algorithms were completed in order to identify feature genes that were highly correlated with RF from the candidate genes in GSE76882 dataset. Specifically, least absolute shrinkage and selection operator (LASSO) analysis was carried out applying glmnet package (v 4.1-2) (30), and it was founded on the idea of using lambda to find significant feature variables and setting the coefficients of less important variables to 0. Through 10-fold cross-validation, genes with non-zero coefficients at the lowest Lambda value in cross-validation were selected as feature genes 1. Boruta was completed based on the Boruta package (v 8.0.0) (31), which designed to find the really important features from a given set of features genes 2. Subsequently, feature genes 1 and feature genes 2 obtained from the above two kinds of machine learning methods were overlapped through the ggvenn package (v 0.1.10) (27), to yield key feature genes.

### 2.7 Expression analysis and receiver operating characteristic analysis

In order to clarify the expression of key feature genes in RF and control samples, the expression of key feature genes was analyzed in GSE76882 and GSE135327 datasets, and comparison of discrepancies between two groups was accomplished through Wilcoxon test. We paid more attention to genes that were differentially expressed between groups (*P* < 0.05) and had consistent expression trends in both datasets, which will be named as candidate biomarkers. Importantly, ROC curves for the candidate biomarkers were plotted in both datasets with the use of the pROC-package (v 1.18.0) (32), in order to assess their ability to distinguish between RF patients and control samples. Genes with area under curve (AUC) values greater than 0.7 in both datasets were identified as biomarkers.

### 2.8 Creation and assessment of nomogram

To estimate diagnostic value of the biomarkers in the clinical setting, nomogram was created in GSE76882 dataset applying the rms package (v 6.5-1) (33). Biomarkers were scored using a nomogram, with each biomarker corresponding to a score. The total score was calculated by summing the scores of all biomarkerss, and the incidence of RF could be inferred based on the total score—the higher the score, the higher the likelihood of RF. To validate the predictive efficacy of the nomogram, the calibration curve was plotted using the regplot package (v 1.1) to reflect the prediction ability of the nomogram model (34). Meanwhile, the Hosmer-Lemeshow (HL) test was performed to determine the discrepancy between predicted and actual values (p > 0.05). The closer the calibration curve was to the diagonal position, the stronger the prediction ability of the nomogram model. Additionally, the ROC curve of the nomogram was drawn using the pROC package (v 1.18.0) to evaluate its diagnostic value, with AUC > 0.7 considered as the model having accuracy (32). Finally, the decision curve analysis (DCA) was plotted using the ggDCA package (https://www.rdocumentation.org/packages/ggDCA/versions/1.1) to assess the clinical practicality of the prediction model.

### 2.9 Gene set enrichment analysis

To explore the signaling pathways involved in the biomarkers, GSEA was performed on the biomarkers in the GSE76882 dataset. Briefly, Spearman correlation coefficients between each biomarker and remaining genes were first computed using the psych package (v 2.2.9) (35), following which these genes were sorted by correlation coefficients in descending order to obtain gene list corresponding to each biomarker. Then, clusterProfiler package (v 4.2.2) was used to complete the GSEA (28), and the reference gene set was “c2.cp.kegg.v7.4.symbols.gmt” in MSigDB database, with *P* < 0.05 and |NES| > 1 as screening criteria for enrichment pathways.

### 2.10 Immunological characterization

VEGF, IL-17, IL-6, IL-8, IL-1Ra, TNF-α, IL-34, and TGF-β were generally regarded as pro-fibrotic factors during the fibrosis process (36–41). To explore the roles of these cytokines in RF, the expression differences of these cytokines between the RF group and the control group were compared via the Wilcoxon test (*P* < 0.05) in the GSE76882 dataset. Next, Spearman correlation analysis was completed to explore the correlation of biomarkers with 28 immune cells and cytokines (*P* < 0.05, |cor| > 0.3).

### 2.11 Recognition of molecular patterns and exploration of biological functions

To determine the possibility of biomarkers guiding molecular subtyping of RF, consensus clustering analysis was performed on 42 RF samples from the GSE76882 dataset using the ConsensusClusterPlus package (v 1.66.0) based on the biomarkers (42), the best clustering was selected by combining the cumulative distribution function. To confirm the dependability of the consensus clustering results, the expression profiles of the identified molecular patterns were subjected to principal component analysis (PCA) using the procmp function in the stats package (v 4.3.2).

Next, DEGs between different molecular patterns were mined through limma package, with screening thresholds of *P* < 0.05 and |log_2_FC| > 1. Following this, these DEGs were incorporated into enrichment analyses exploring GO function and KEGG pathways associated with these genes, which were completed using clusterProfiler (*P* < 0.05). Furthermore, the pathway enrichment scores of samples from different molecular patterns were calculated by gene set variation analysis (GSVA) applying “hallmark pathway genes set” in MSigDB database as the background gene set, followed by comparison of the differences in the biological pathways between different molecular patterns by Wilcoxon (*P* < 0.05).

Finally, ssGSEA was applied to calculate the infiltration score of 28 immune cells in 42 RF samples, and Wilcoxon test was utilized to accomplish comparison of discrepancies in infiltration score in different molecular patterns (*P* < 0.05). In addition, differences in cytokines in different molecular patterns were also emphasized.

### 2.12 Molecular regulation analysis

The microRNAs (miRNAs) regulating the biomarkers were predicted applying the Diana_microtT (https://dianalab.e-ce.uth.gr/microt_webserver/) and miRDB (https://mirdb.org). Subsequently, upstream long non-coding RNAs (lncRNAs) of miRNAs were retrieved by accessing miRNet database (https://www.mirnet.ca). Based on above results, lncRNA-miRNA-mRNA (biomarker) network was generated with the use of Cytoscape software. Additionally, transcription factors (TFs) targeting biomarkers were retrieved from ChEA3 (https://maayanlab.cloud/chea3) database. TFs with *P* < 0.05 were selected for visualization.

### 2.13 Drug prediction and molecular docking

To further screen potential drugs for the treatment of RF, biomarkers were entered into the DGIdb database (https://dgidb.org/) to retrieve drugs targeting the biomarkers. Crystal structure of the protein corresponding to the biomarker was retrieved using Protein Data Bank (PDB) database (https://www.rcsb.org/), and 3D structure of the drug was retrieved using PubChem database (https://pubchem.ncbi.nlm.nih.gov/). Then, molecular docking was accomplished and binding energy was obtained with the help of Autodock software. It was generally accepted that binding energy ≤ -5 kcal/mol was considered to have a strong binding capacity.

### 2.14 Animal experiments verification

#### 2.14.1 Animal experiments protocol

Ten specific pathogen free (SPF) male Sprague Dawley (SD) rats (42–48 days old, weighing 200-250g) were purchased from Beijing Weilitonghua Laboratory Animal Technology Co., Ltd. (Animal Production License No.: SCXK (Jing) 2021-0006). The experimental animals were housed in the SPF-level animal room provided by the Experimental Animal Center of Nanjing University of Chinese Medicine (Animal Use License No.: SYXK (Su) 2023-0077), and were fed with SPF-level maintenance feed and given free access to water. This study adheres to the guidelines of the National Institutes of Health for the care and use of laboratory animals. The experimental protocols comply with the relevant ethical regulations and requirements for animal experiments and have been approved by the Animal Ethics Committee of Nanjing University of Chinese Medicine (Approval No.: ACU231205). After one week of adaptive feeding, all SD rats were randomly divided into two groups, with five rats in each group: the control group (Ctrl group) (sham operation + standard feed diet), and the RF model group (Model group) (5/6 nephrectomy + 1% high choline diet). The RF model was based on our previous research, where the combination of a 1% high choline diet with a common nephropathy model could further aggravate the progression of RF (43, 44). The rats in both groups were euthanized 8 weeks after modeling, and the relevant specimens were retained for detection and analysis before euthanasia.

Ten male C57BL/6J mice aged 8–10 weeks (weighing 18-22g) were purchased from Zhejiang Weitong Lihua Laboratory Animal Technology Co., Ltd. (Animal Production License No.: SCXK (Su) 2022-0006) and were raised under the same conditions as described previously. This experiment also adhered to the guidelines of the National Institutes of Health for the care and use of laboratory animals and was approved by the Ethics Committee of the Affiliated Hospital of Nanjing University of Chinese Medicine (Approval Document No.: 2023 DW-016-01). All mice were randomly divided into two groups: the control group (Ctrl group) (sham operation), and the RF model group (Model group) (unilateral ureteral obstruction). The method for establishing the unilateral ureteral obstruction (UUO) RF model was the same as in our previous study (45). Fourteen days after modeling, all mice were anesthetized by intraperitoneal injection of 3% pentobarbital sodium (0.5 ml/100 g), relevant specimens were retained before euthanasia.

#### 2.14.2 Detection of renal function related indicators

The levels of serum creatinine (SCR) and blood urea nitrogen (BUN) in rats were detected using a Dimension EXL200 automatic biochemical analyzer (Siemens, Germany).

#### 2.14.3 Renal tissue pathological staining

Renal tissue specimens fixed in 4% paraformaldehyde solution were taken. After routine dehydration, clearing, wax immersion and embedding, paraffin sections of about 4 μm were made, with the section direction perpendicular to the long axis of the kidney. The renal tissue paraffin sections were stained with hematoxylin-eosin (HE), Masson and periodic acid-Schiff (PAS) to observe the changes in renal cortical tissue structure and collagen deposition by using a light microscope (magnification×200, Nikon Eclipse Ni-U, Japan).

#### 2.14.4 Immunohistochemistry

After dewaxing paraffin sections to water, antigen retrieval was performed at high temperature and high pressure (antigen retrieval solution: pH 9.0 EDTA). Then, endogenous peroxidase was blocked with 3% hydrogen peroxide, and the sections were circled with a histochemical pen. After incubation with 10% goat serum at room temperature for blocking, 100 μl of the working solution of CD74 (1:200) (Affinity, China), AGR2 + AGR3 (1:300) (since AGR3 and AGR2 are highly related homologous genes, we chose the AGR2 + AGR3 antibody for subsequent detection, Abcam, USA), and SYT11 (1:200) (Proteintech, China) primary antibodies were added to each tissue section and incubated at 4°C overnight. The next day, after rewarming and washing, 100 μl of the working solution of the secondary antibody corresponding to the species of the primary antibody was added to each section and incubated at 37°C for 45 minutes. After washing, 100 μl of fresh DAB was added to each section. Color development was observed under a microscope and stopped with tap water. Sections were counterstained with hematoxylin, differentiated with alcohol - hydrochloric acid, and blued. Then, they were dehydrated with gradient alcohol, dried, and sealed with an eco - friendly mounting medium (46). Finally, the expression and localization of related proteins in kidney cortical tissue were observed at ×200 magnification using a Nikon Eclipse Ni - U microscope and images were collected (positive expression was brown or dark brown).

#### 2.14.5 Immunofluorescence

After dewaxing paraffin sections to water, antigen retrieval was performed under high temperature and high pressure (antigen retrieval solution: EDTA, pH 9.0). Circles were drawn on the sections with a histochemical pen, and then 10% donkey serum was added for blocking. 50-100 μl of the primary antibody working solution (CD74 (1:200), AGR2 + AGR3 (1:200), and SYT11 (1:200) primary antibody) was dropped onto each section and incubated at 4°C overnight. The next day, after rewarming and washing, 1 μl of Alexa Fluor^®^ 488 donkey anti-rabbit IgG (H+L) secondary antibody solution was added to 400 μl of TBST to prepare the secondary antibody working solution. 50-100 μl (depending on the size of the tissue) of the secondary antibody working solution was dropped onto each section and incubated at 37°C for 45 minutes. After routine TBST washing, 50-100 μl of DAPI working solution was dropped onto each section, and the nuclei were stained in the dark for 5 minutes. Then, the sections were washed with TBST again and sealed with a fluorescence mounting medium. Finally, the fluorescence intensity and localization of the related proteins in the kidney cortical tissue were observed using a fluorescence microscope (magnification×400, Nikon Eclipse C1, DS-U3, Japan), and images were collected and analyzed.

#### 2.14.6 Western blot

Equal amounts of 20 µg proteins of each group were loaded and separated by 10% sodium dodecyl sulfate-polyacrylamide gel electrophoresis (SDS-PAGE). The separated proteins were transferred to activated polyvinylidene difluoride (PVDF) membranes by wet transfer method and then blocked. The membranes were incubated with primary antibodies CD74 (1:500), AGR3 (1:500), and SYT11 (1:500) overnight at 4°C, and then incubated with the corresponding secondary antibodies at room temperature for 1 hour. The bands were developed using ECL chemiluminescent solution and photographed using the ChemiDocTM XRS+ with Image Lab Software (Bio-Rad, Hercules, CA, USA) chemiluminescence system (47). The gray values of the target bands were determined using Image J (1.52a) software. The gray value of β-tubulin (1:3000) (Proteintech, China) was used as an internal reference.

### 2.15 Statistical analysis

R program (v 4.2.0) was utilized for bioinformatic statistics analysis. The scoring tests between two groups were analyzed using Wilcoxon-test. Discrepancies were deemed statistically meaningful when *P* value was below 0.05.

Animal experiments statistical analysis was performed using SPSS 19.0 software. All experimental data were expressed as mean ± standard deviation (SD). The Student’s t-test was used for comparison between groups. The *P* value < 0.05 was considered statistically significant.

## 3 Results

### 3.1 Identification of 692 key modular genes associated with macrophage and lactate metabolism

**Figure 1:**
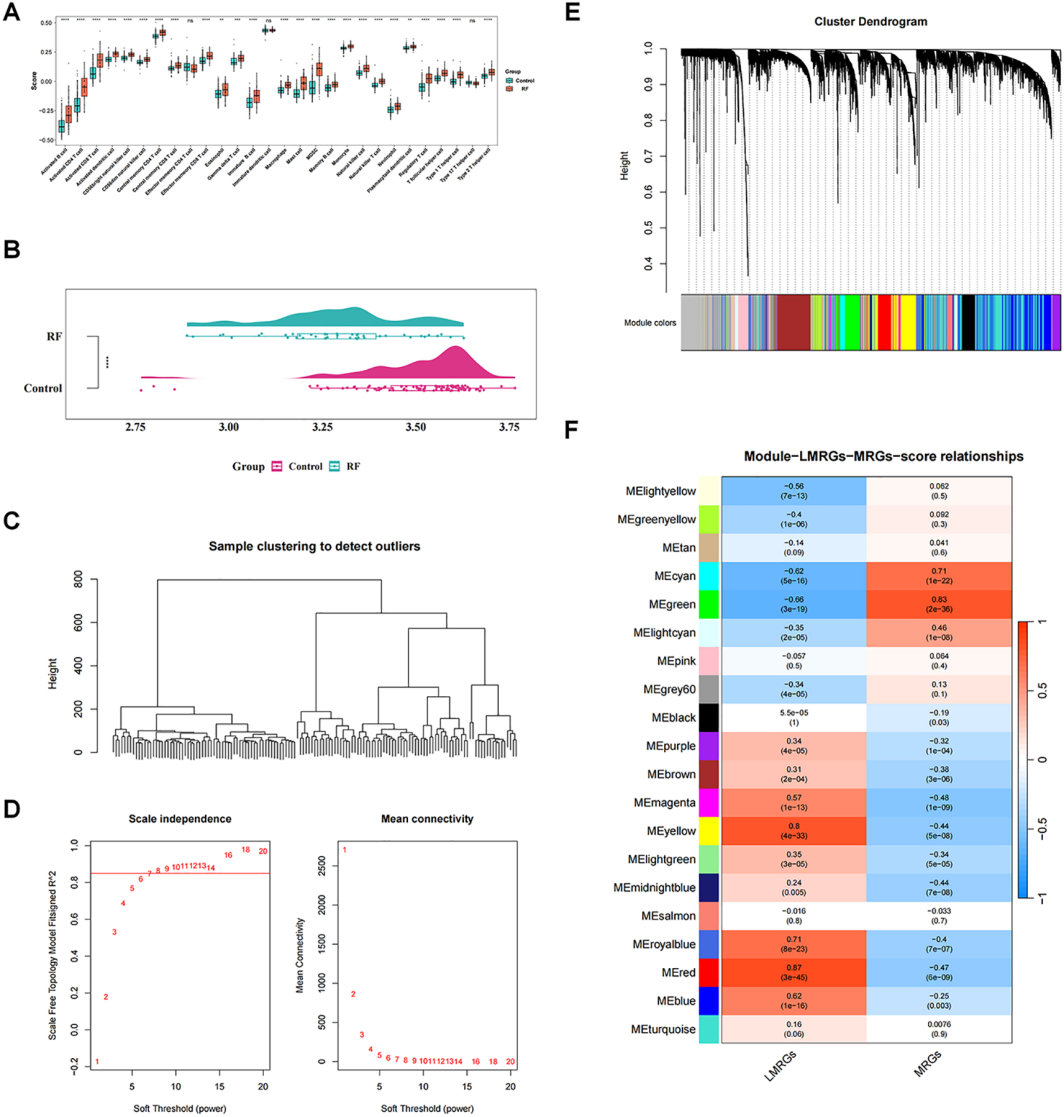
Identification of key modular genes associated with macrophage and lactate metabolism. **(A)** Differences in immune cells between renal fibrosis (RF) and control samples. ns, not significant; **P value < 0.01; ***P value < 0.001; ****P value < 0.0001. **(B)** Single-sample gene set enrichment analysis (ssGSEA) score raincloud plots of lactate metabolism-related genes (LMRGs). **(C)** Sample hierarchical clustering plot. Each branch in the clustering tree represents a sample, and the vertical coordinate represents the Euclidean distance of sample expression levels. **(D)** Selection of soft threshold. **(E)** Identification of co-expression modules. **(F)** Correlation heatmap between modules and ssGSEA. The darker the color, the higher the correlation. Red indicates positive correlation, and blue indicates negative correlation. The number in each cell represents the correlation and significance.

In GSE76882 dataset, the ssGSEA algorithm revealed that 25 immune cells including macrophages were markedly distinct between RF and control groups (*P* < 0.05), and all were highly expressed in RF group (**Figure 1A**). Moreover, we noted that RF group had significantly lower LMRGs score in comparison to the controls (*P* < 0.05) (**Figure 1B**).

With respect to WGCNA, no significant outliers between the samples were observed through cluster analysis, revealing excellent clustering (**Figure 1C**). Based on the fact that R^2^ was equal to 0.85 and the mean connectivity tended to 0, we choose the optimal β-value of 8 to satisfy the scale-free topology of the network (**Figure 1D**). Subsequently, a clustering tree diagram was constructed by gene correlation and adjacency, and 21 co-expressed gene modules were acquired (**Figure 1E**). Correlation analysis showed that MEgreen had significantly and strongly correlations with both macrophage score and LMRGs score (|cor| > 0.65, *P* < 0.05), with MEgreen having the highest positive correlation with macrophage score (cor = 0.83, *P* < 0.05) and the most that negative correlation with LMRGs score (cor = -0.66, *P* < 0.05) (**Figure 1F**). Therefore, 692 genes contained in MEgreen were considered as key modular genes highly correlated with macrophage score and LMRGs score.

### 3.2 Revealing the biological functions and PPI of 384 candidate genes

**Figure 2:**
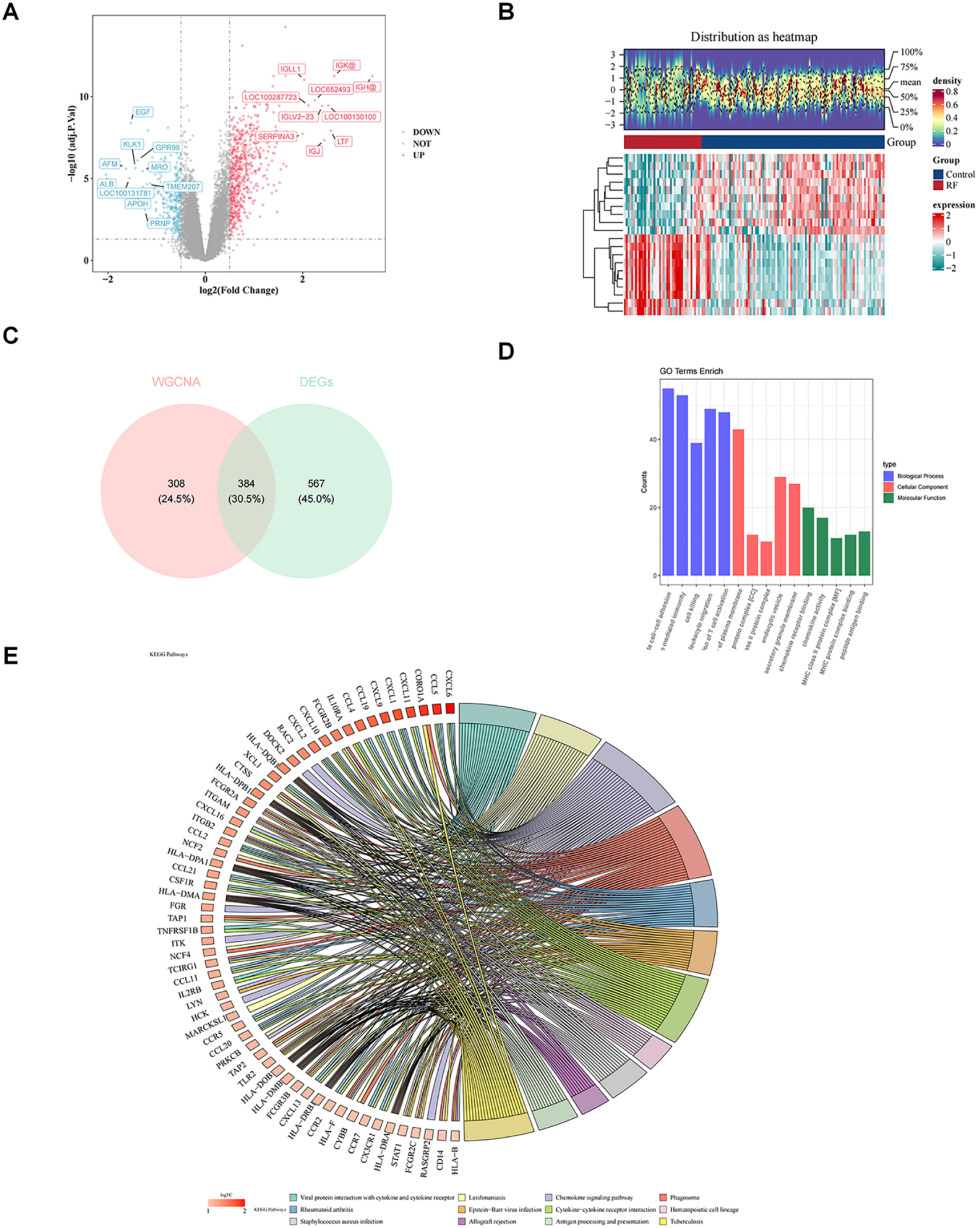
Revealing the biological functions of candidate genes. **(A)** Volcano plot of differentially expressed genes. **(B)** Heatmap of differentially expressed genes. **(C)** Venn diagram for identification of candidate key genes. Pink represents genes related to macrophage-related genes (MRGs) and LMRGs, while blue represents genes related to differentially expressed genes (DEGs). **(D)** Bar chart of Gene Ontology (GO) enrichment. The pathways shown are biological processes (BP), cellular components (CC), and molecular functions (MF), with the top 5 pathways ranked by significance (*P* value from smallest to largest). **(E)** Kyoto Encyclopedia of Genes and Genomes (KEGG) enrichment chord diagram. The color of the genes on the left represents the logFoldChange(FC) of the genes, and the different color bands on the right represent different pathways.

In total, 951 DEGs were identified between RF and control groups in GSE76882 dataset through differential expression analysis, including 648 up-regulated genes 303 down-regulated genes in RF samples (**Figure 2A**). Heat map demonstrated top 10 up-regulated genes and top 10 down-regulated genes (**Figure 2B**). Thereafter, intersecting genes of 692 key module genes and 951 DEGs were collected, resulting in 384 candidate genes (**Figure 2C**).

Subsequent enrichment analysis of these 384 candidate genes yielded 875 GO entries and 67 KEGG pathway (*P* < 0.05). In GO-BP term, candidate genes were mainly engaged in “leukocyte mediated immunity”, “cell killing”, “leukocyte migration”, “regulation of T cell activation”, etc (**Figure 2D** **)**. In GO-CC, candidate genes were mainly localized to “MHC class II protein complex”, “endocytic vesicle”, “secretory granule membrane”, etc (**Figure 2D** **)**. GO-MF associated with candidate genes included “chemokine receptor binding”, “MHC protein complex binding”, “peptide antigen binding”, etc (**Figure 2D** **)**. Furthermore, KEGG analysis revealed that candidate genes were engaged in “chemokine signaling pathway”, “cytokine-cytokine receptor interaction”, etc (**Figure 2E**).

PPI network of 384 candidate genes contained 339 nodes and 6046 edges, in which genes such as ACKR4, CCL11, and CCL19 had stronger interactions with the remaining genes (**Supplementary Figure S1**).

### 3.3 Screening and diagnostic value of AGR3, CD74, and SYT11 in RF

**Figure 3:**
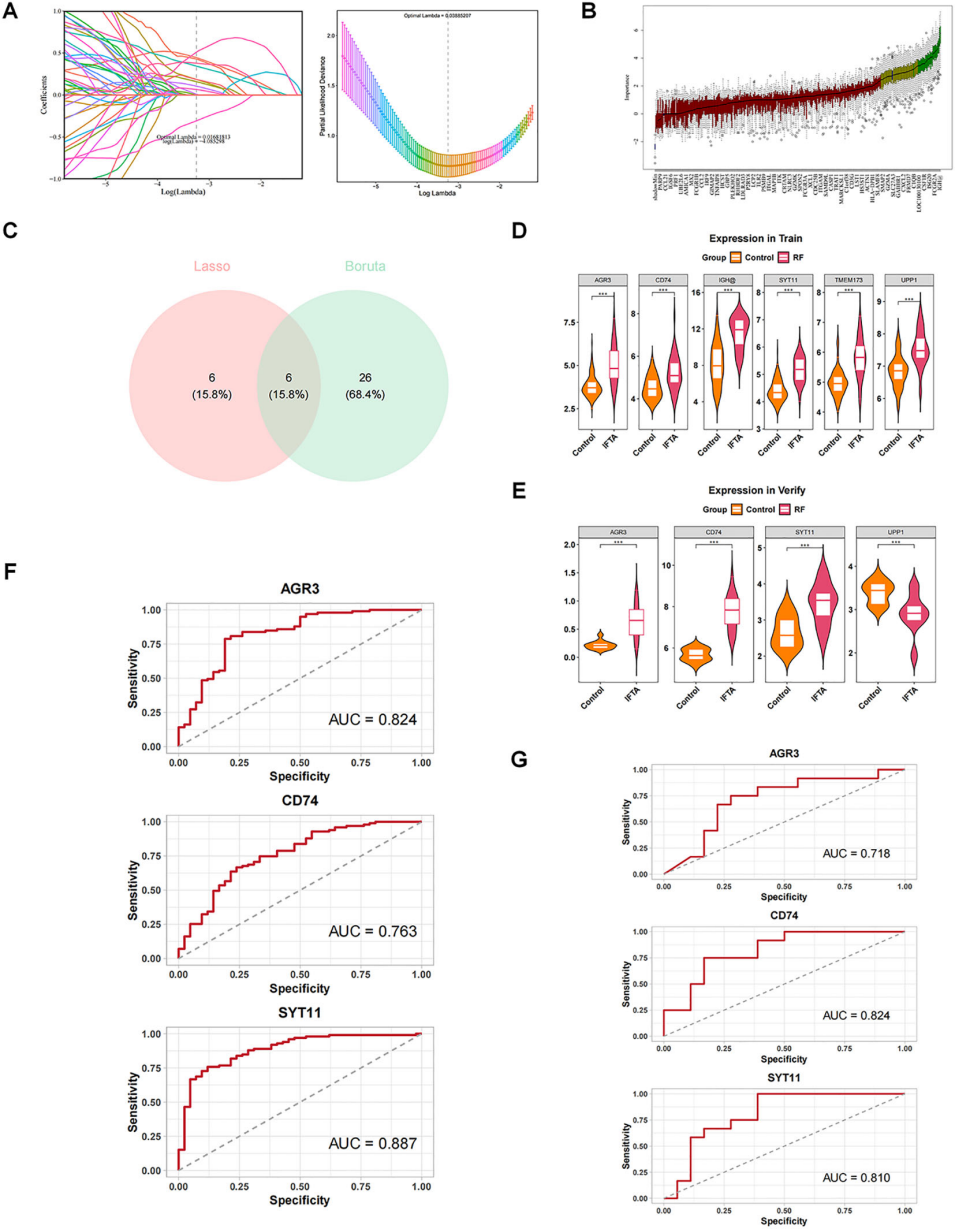
Screening and diagnostic value of AGR3, CD74, and SYT11 in RF. **(A)** Least absolute shrinkage and selection operator (LASSO) regression analysis screening and cross-validation. **(B)** Boruta analysis screening results. Blue box: The minimum, average, and maximum Z-values of the shadow property; Red: rejection feature; Yellow: features to be confirmed; Green box: Confirmed feature. **(C)** Intersection of the two algorithms to obtain key feature genes. Pink represents genes confirmed by Lasso regression, and blue represents genes confirmed by Boruta as relevant. **(D)** Expression of key feature genes in the training set. **(E)** Expression of key feature genes in the validation set. **(F)** Receiver operating characteristic (ROC) analysis of candidate key genes in the training set. **(G)** ROC analysis of candidate key genes in the validation set.

Depending on the expression of 384 candidate genes in GSE76882, machine learning was performed in combination with the LASSO and Boruta algorithms to screen the feature genes. With LASSO regression analysis, 12 feature genes associated with RF were identified based on lambda._min_ value of 0.03885 (**Figure 3A**). Meanwhile, Boruta algorithm showed 32 feature genes based on the importance of each feature (**Figure 3B**). The Venn diagram demonstrated six key feature genes (IGH, UPP1, TMEM173, CD74, SYT11, and AGR3) by taking the intersection of feature genes in two machine learning methods (**Figure 3C**).

Expression analyses showed that AGR3, CD74, and SYT11 had the same expression trend in GSE76882 and GSE135327 datasets and were markedly different between RF and control groups (*P* < 0.05), with higher expression in the RF group (**Figures 3D, E**). Moreover, it could be observed from the ROC curves that the AUC values of these three genes were greater than 0.07 in two datasets, implying that they exhibited a high accuracy in the diagnosis of RF (**Figures 3F, G**). Therefore, AGR3, CD74, and SYT11 were considered as biomarkers associated with macrophage and lactate metabolism in RF.

### 3.4 Building an effective nomogram for diagnosing RF

**Figure 4:**
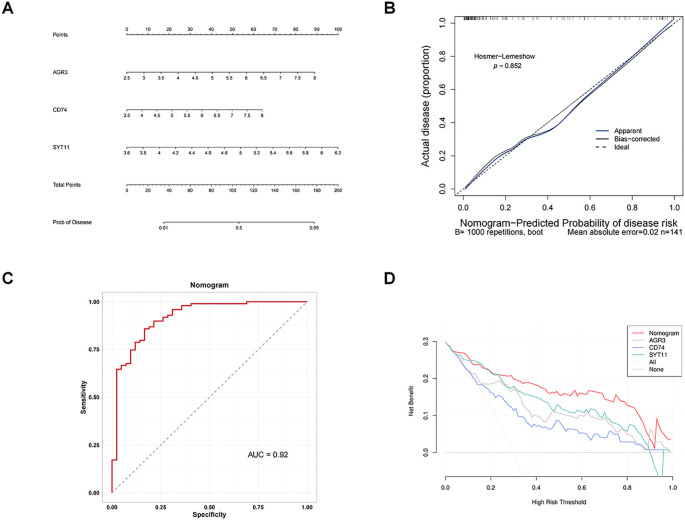
Building an effective nomogram for diagnosing RF. **(A)** Nomogram. **(B)** Calibration curve of the nomogram model. **(C)** ROC curve of the nomogram. **(D)** Decision curve analysis (DCA) curve of the nomogram.

By integrating the expression of three biomarkers in GSE76882 dataset, we created a nomogram to predict the risk of RF (**Figure 4A**). Each biomarker corresponded to a score, and the individual scores were summed to obtain a total score; the higher the total score, the higher the likelihood of RF. There was no difference between the predicted and true values in calibration curve and the value of AUC in the ROC curve was 0.92 (**Figures 4B, C**), meaning that there was a high accuracy in predicting RF using the nomogram. In addition, DCA results revealed that net benefit value of the nomogram was higher than that of individual biomarkers (**Figure 4D**), suggesting that the nomogram has potential clinical applications.

### 3.5 Elucidating the biological mechanisms of biomarkers

**Figure 5:**
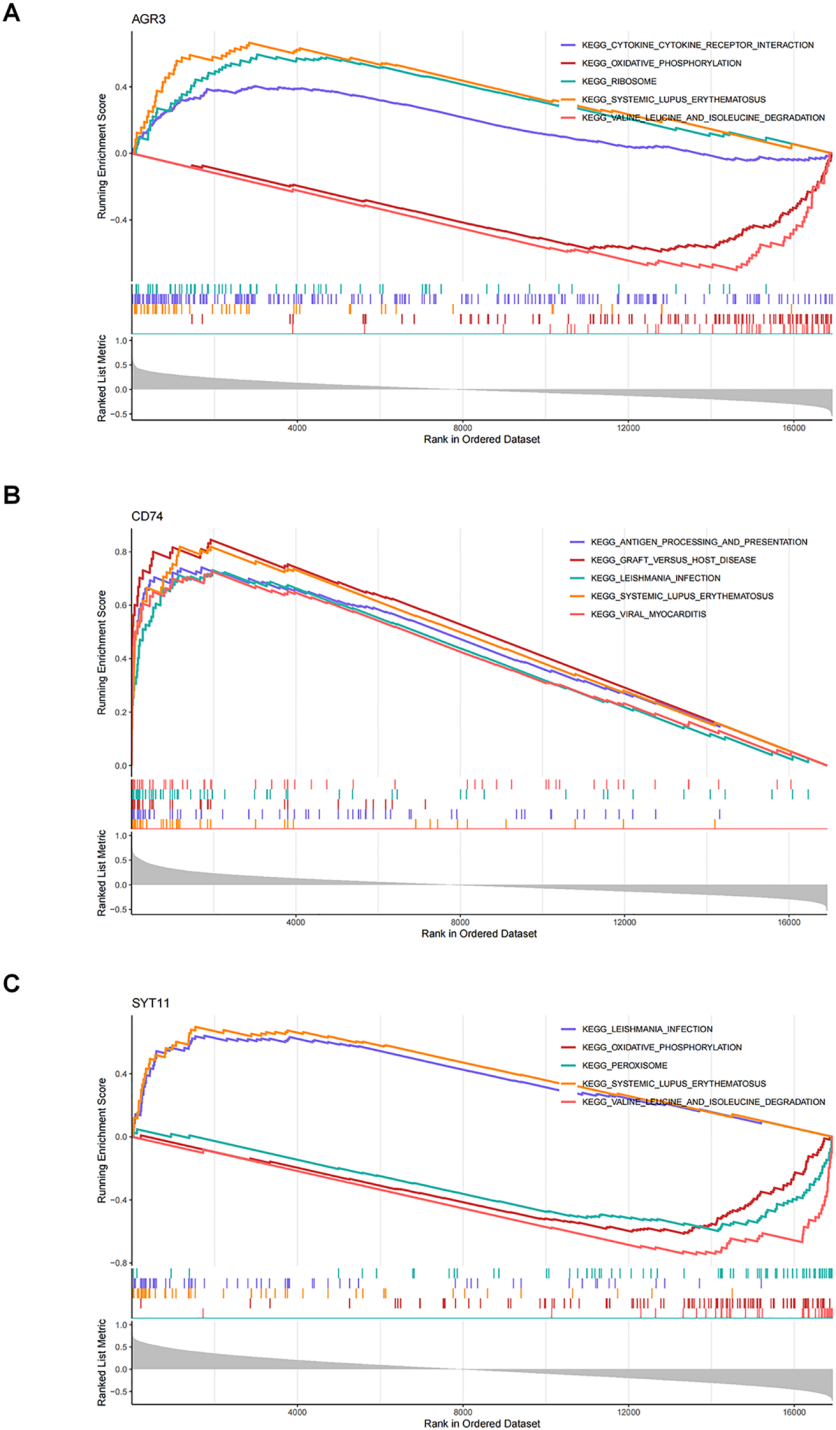
Elucidating the biological mechanisms of biomarkers. **(A)** Gene set enrichment analysis (GSEA) of AGR3. **(B)** GSEA of CD74. **(C)** GSEA of SYT11.

GSEA was completed in the GSE76882 dataset to elucidate the signaling pathways involved in the three biomarkers. The results showed that the three biomarkers were collectively enriched to 50 signaling pathways, comprising “oxidative phosphorylation”, “Toll-like receptor signaling pathway”, “T cell receptor signaling pathway”, “JAK-STAT signaling pathway”, “P53 signaling pathway”, “phenylalanine metabolism”, etc (**Supplementary Tables S2–S4**). The top 5 pathways were selected for visualization based on significance ranking (**Figures 5A–C**). The above findings suggested that biomarkers influenced the pathological process of RF by participating in pathways related tocellular metabolism, energy, immune system, and cell transduction.

### 3.6 Uncovering the relationship between biomarkers and immune profiles

**Figure 6:**
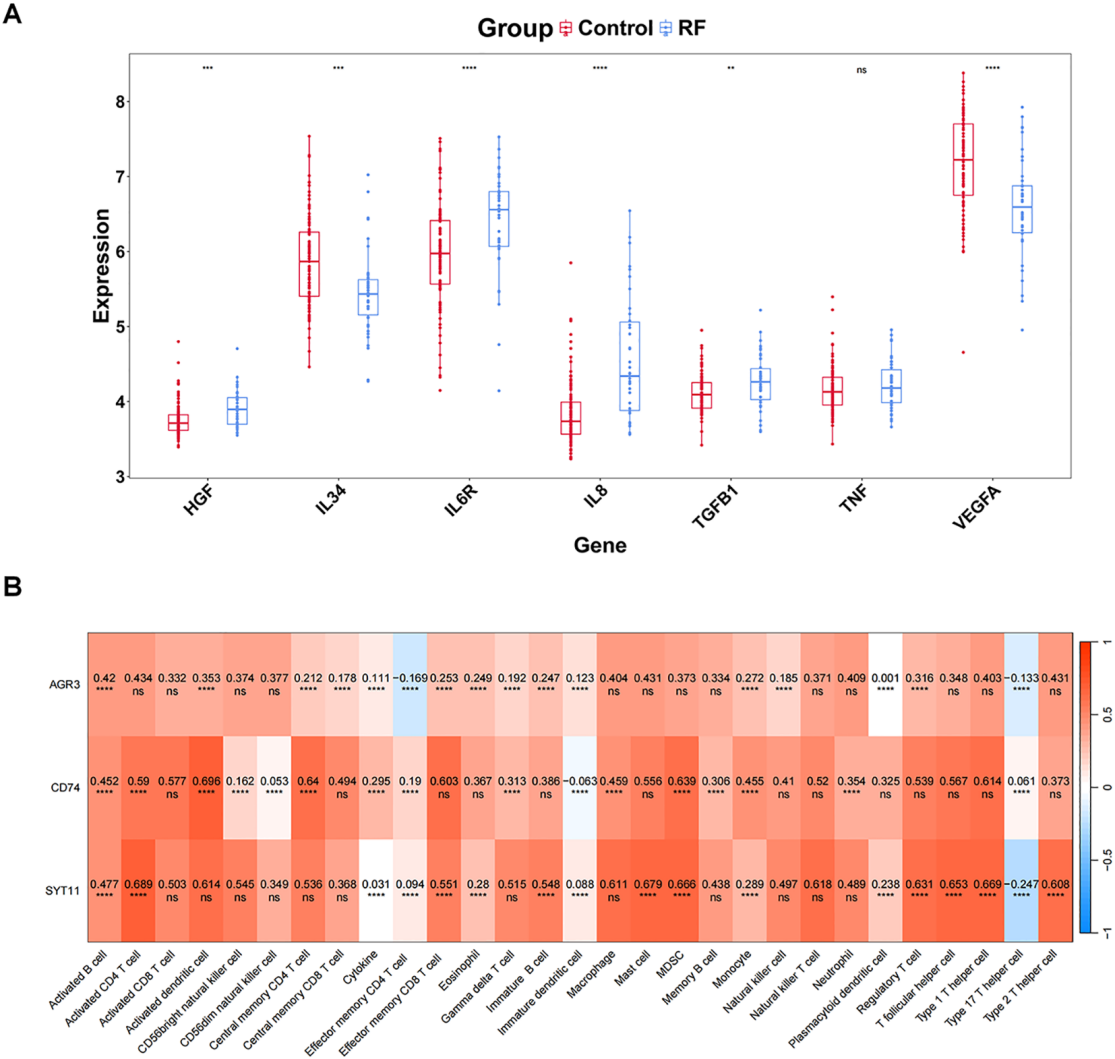
Uncovering the relationship between biomarkers and immune profiles. **(A)** Differential expression of cytokines/inflammatory factors. **(B)** Correlation between key genes and all immune features (immune cells, cytokines/inflammatory factors). The values in the cells represent the correlation. ns, P value ≥ 0.05; **P value < 0.01; ***P value < 0.001; ****P value < 0.0001.

Comparison of eight cytokines between RF and control groups in GSE76882 dataset showed significant differences between groups for VEGFA, HGF, IL8, IL6R, IL34, and TGFB1 (*P* < 0.05), with HGF, IL6R, IL8, and TGFB1 being highly expressed in RF samples, and IL34 and VEGFA showing the opposite trend (**Figure 6A**).

Subsequently, the eight cytokines as a whole (Cytokine) were analyzed for correlation with the biomarkers, and the results indicated a remarkable positive association between Cytokine and three biomarkers (*P* < 0.05) (**Figure 6B**). Furthermore, we noted the highest significant positive correlations between AGR3 and activated B cells (cor = 0.420 and *P* < 0.001), between CD74 and activated dendritic cell (cor = 0.696 and *P* < 0.001), and between SYT11 and activated CD4 T cell (cor = 0.689 and *P* < 0.001) (**Figure 6B**).

### 3.7 Exploration of subtypes associated with biomarkers

**Figure 7:**
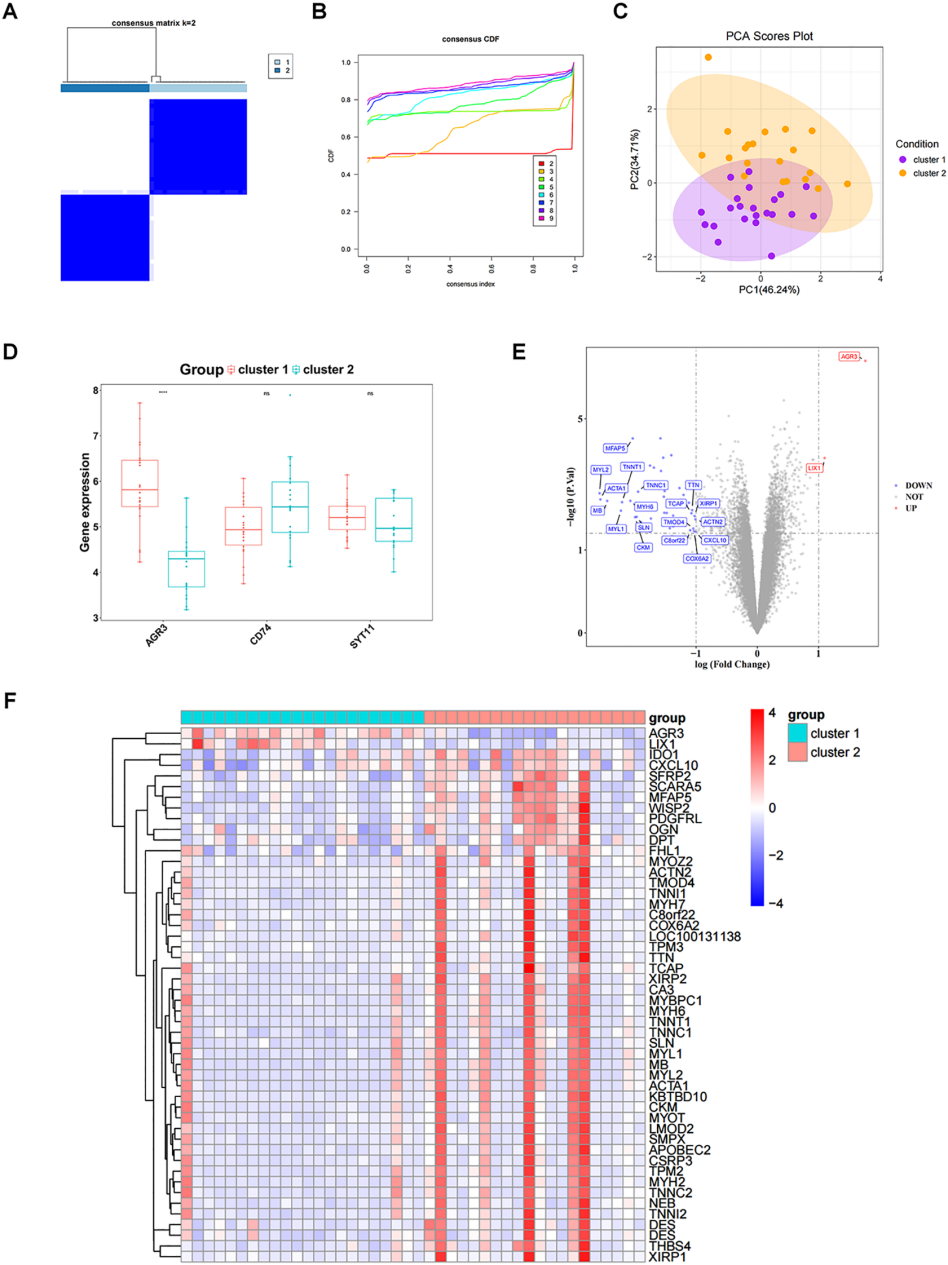
Exploration of subtypes associated with biomarkers. **(A)** Clustering effect diagram of two subtypes of RF samples. **(B)** Consensus cumulative distribution function. **(C)** Principal component analysis (PCA) analysis of expression profiles of different molecular patterns. **(D)** Box plot of key gene expressions in different molecular patterns. **(E)** Volcano plot of DEGs_2 between different molecular patterns. DEGs_2 represents the differentially expressed gene set between cluster1 and cluster2. **(F)** Heatmap of DEGs_2 between different molecular patterns. ns, P value ≥ 0.05; ****P value < 0.0001.

Consistent clustering analysis was performed according to the expression of three biomarkers in RF samples from the GSE76882 dataset, yielding two RF-related subtypes (cluster 1 and cluster 2) (**Figures 7A, B**). PCA results indicated a superior differentiation between cluster 1 and cluster 2 (**Figure 7C**). Notably, the expression of AGR3 was significantly higher in cluster 1 compared to cluster 2 (**Figure 7D**).

**Figure 8:**
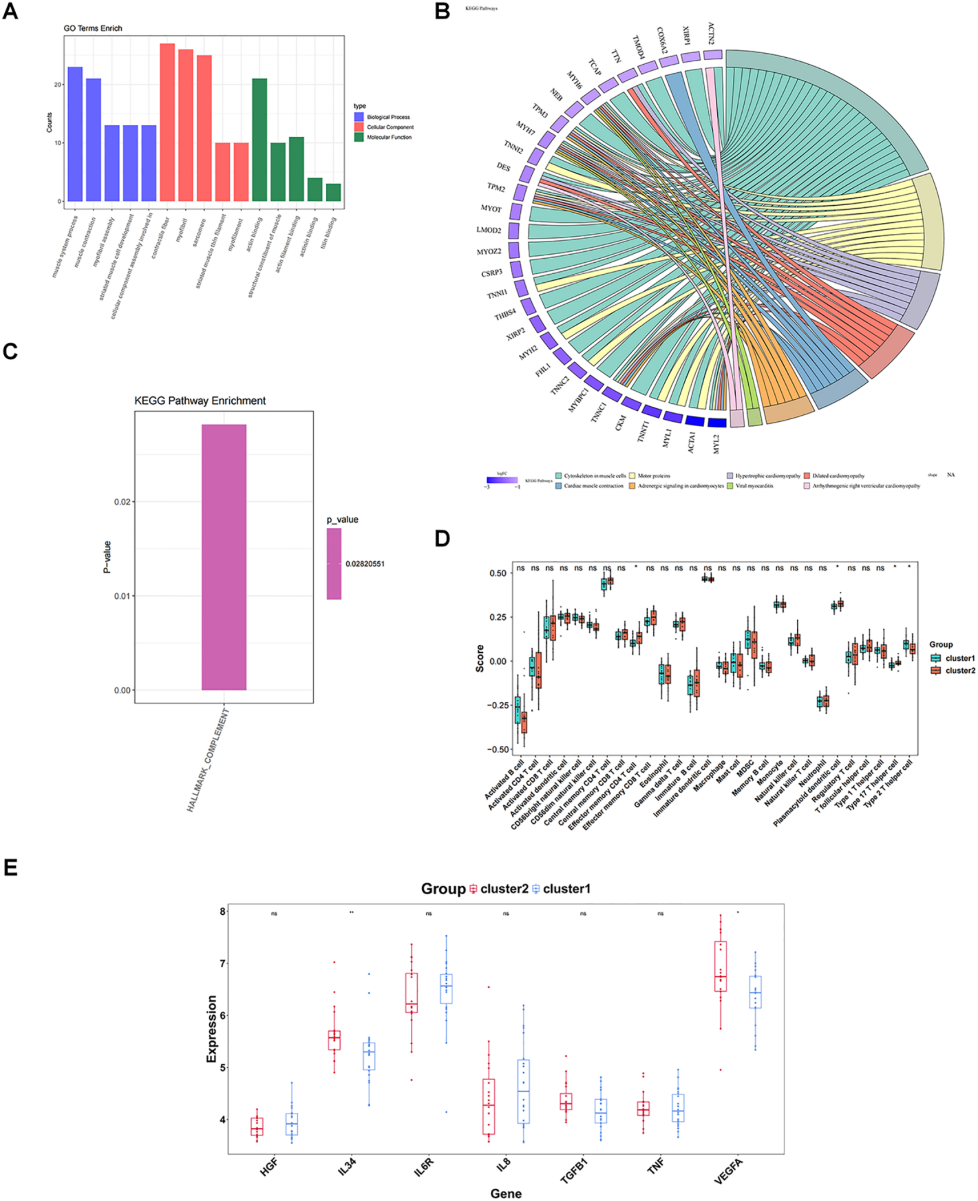
Enrichment analysis and immune characteristic differences of different subtypes. **(A)** GO enrichment between different molecular patterns. The pathways shown are the top 5 pathways of BP, CC, and MF in order of significance (*P* value from smallest to largest). **(B)** KEGG enrichment between different molecular patterns. The color of the genes on the left represents the logFC of the genes, and the different color bands on the right represent different pathways. **(C)** Differences in enriched pathways between different molecular patterns; **(D)** Differences in immune cells between different molecular patterns; **(E)** Differences in cytokines/inflammatory factors between different molecular patterns. ns, P value ≥ 0.05; *P value < 0.05; **P value < 0.01.

The gene expression matrix was further compared between cluster 1 and cluster 2, yielding 50 DEGs, including two up-regulated genes and 48 down-regulated genes in cluster 1 (**Figures 7E, F**). These 50 DEGs were enriched and analyzed, yielding 71 GO entries and 8 KEGG pathways (*P* < 0.05). With respect to GO, the entries were mainly related to “muscle system process”, “muscle contraction”, “myofibril assembly”, “contractile fiber”, “myofibril”, “actin binding”, and so on (**Figure 8A**). KEGG analysis elucidated that these DEGs were engaged in “Cytoskeleton in muscle cells”, “Motor proteins”, “Hypertrophic cardiomyopathy”, etc (**Figure 8B**). In addition, GSVA results showed a significant difference in one pathway (Hallmark Complement) between cluster 1 and cluster 2 (**Figure 8C**).

Finally, four of the 28 immune cells (effector memory CD4 T cells, plasmacytoid dendritic cell, type 17 T helper cell, and type 2 T helper cells) could be noticed to be remarkably distinct between cluster 1 and cluster 2 (*P* < 0.05) (**Figure 8D**). Two cytokines, IL34 and VEGFA, were also remarkably distinct between cluster 1 and cluster 2 (*P* < 0.05), and all were highly expressed in cluster 2 (**Figure 8E**).

### 3.8 Potential regulatory mechanisms of biomarkers

**Figure 9:**
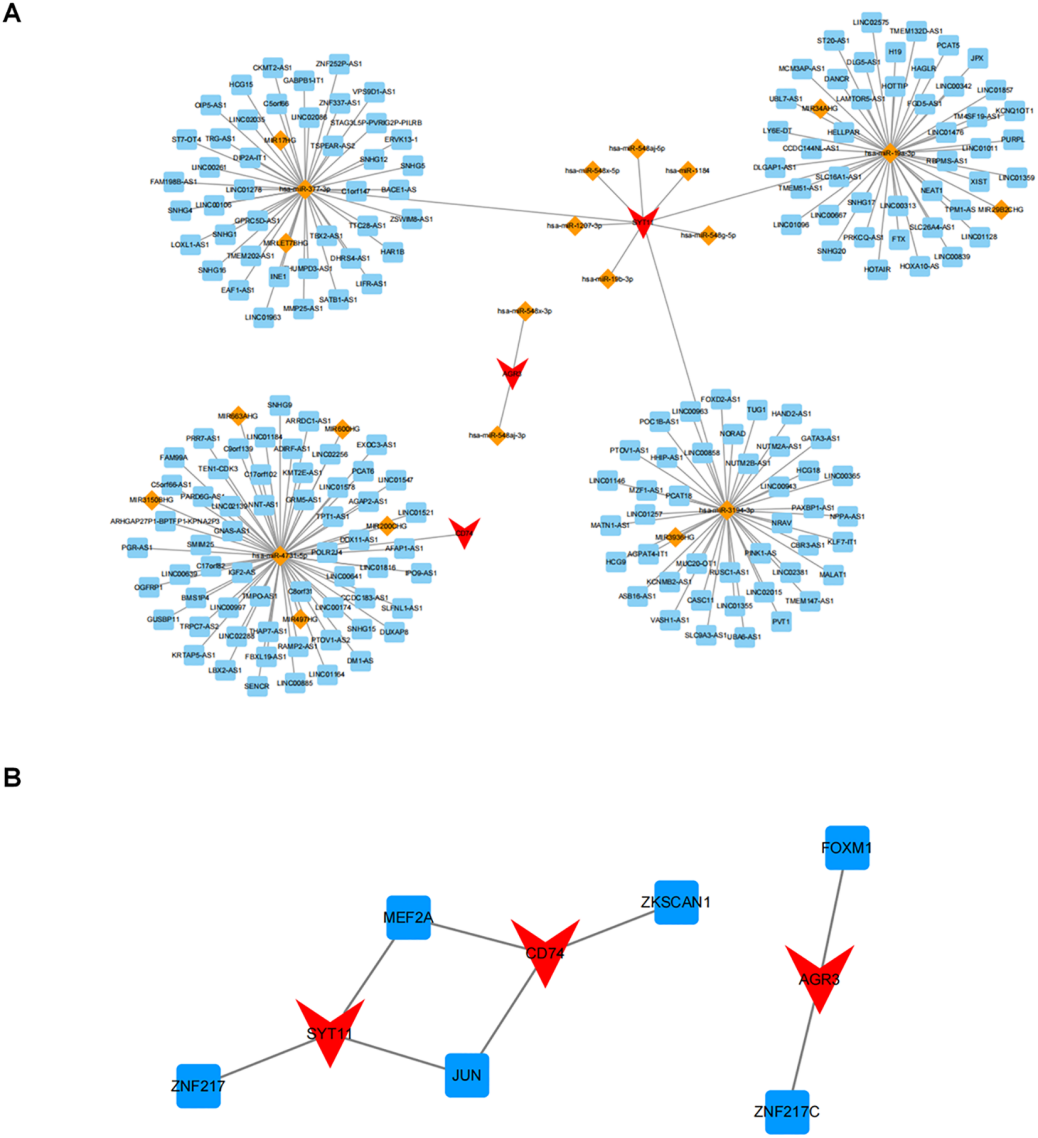
Potential regulatory mechanisms of biomarkers. **(A)** Relationship network of lncRNA-miRNA-key genes. Red represents key genes, orange represents miRNA, and blue represents lncRNA; **(B)** Relationship network of differentially expressed transcription factors (TF) and key genes. Red represents genes and blue represents TF.

A lncRNA-miRNA-mRNA network comprising 20 miRNAs, 189 lncRNAs, and three biomarkers was created by predicting public databases (**Figure 9A**). Multiple relationship pairs could be found in the network, e.g., hsa-miR-548x-3p and hsa-miR-548aj-3p were regulators of AGR3, as well as multiple lncRNAs (PCAT6, POLR2J4, SMIM25, etc.) could co-regulate CD74 through hsa-miR-4731-5p.

In addition, a search of the ChEA3 database applying threshold of *P* < 0.05 yielded six TFs, in which MEF2A and JUN were co-regulators of SYT11 and CD74, and FOXM1 and ZNF217C could regulate AGR3 (**Figure 9B**).

### 3.9 Binding of biomarkers to potential drugs

**Figure 10:**
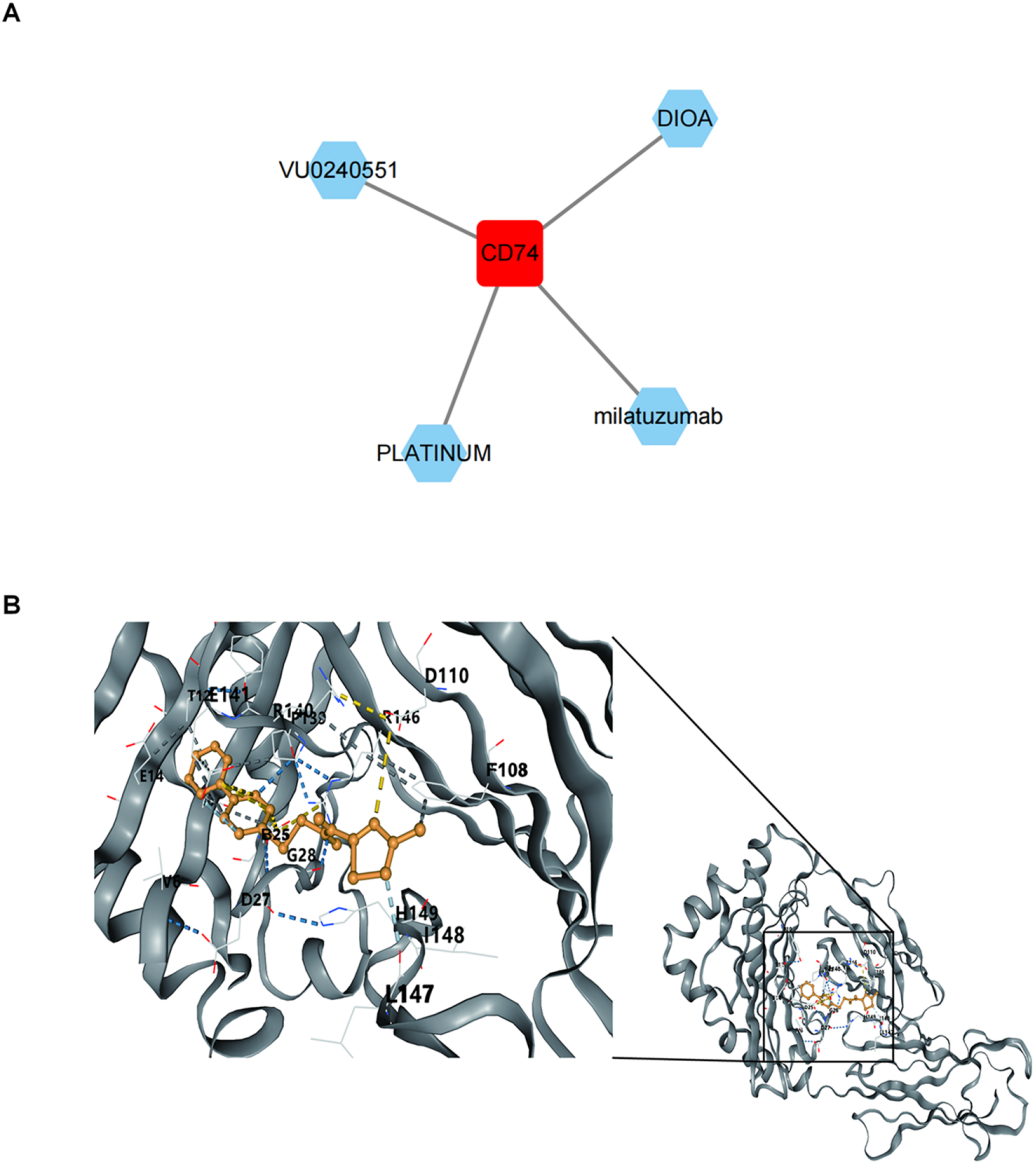
Binding of biomarkers to potential drugs. **(A)** Drug-key gene CD74 relationship network. Red represents key gene CD74 and green represents predicted drug names. **(B)** Molecular docking results of key gene CD74 and small molecule drug VU0240551.

Four potential drugs targeting CD74 were retrieved by thorough analysis of the DGidb database, namely VU0240551, DIOA, milatuzumab, and Platinum (**Figure 10A**). Unfortunately, no drugs targeting SYT11 and AGR3 were retrieved. We chose VU0240551 for subsequent molecular docking because of the highest interaction score (13.12) between VU0240551 and CD74. The results indicated that binding energy between VU0240551 and CD74 was -8.0 kcal/mol, implying a strong affinity (**Figure 10B**).

### 3.10 Animal experiments verification

**Figure 11:**
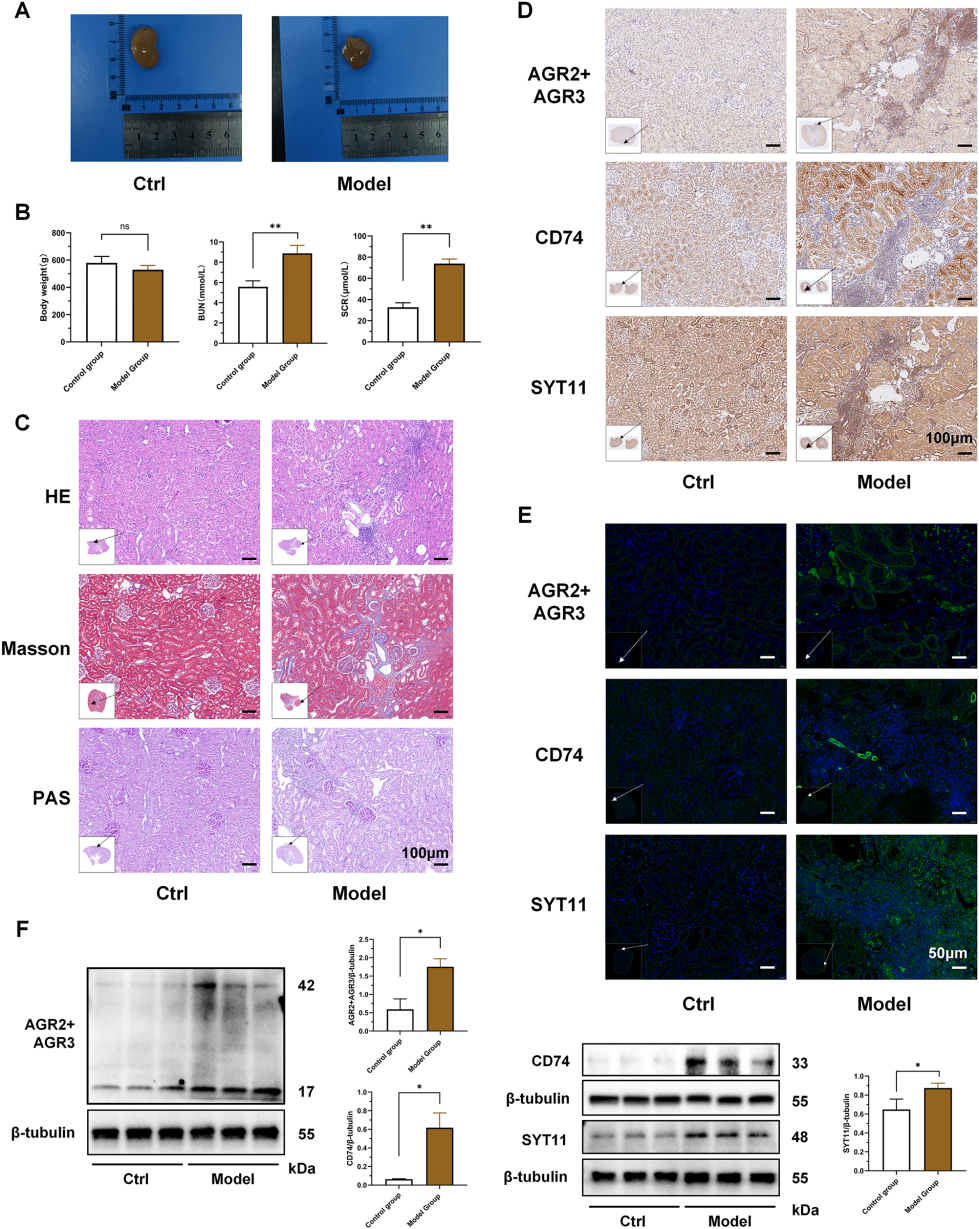
Validation of key biomarker expression in animal experiments. **(A)** Representative kidneys of rats in the two groups at the time of kidney tissue collection. **(B)** Comparison of body weight and renal function between the two groups of rats. **(C)** HE, Masson and PAS pathological staining of kidneys in the two groups of rats. The black arrow indicates the area of this field of view in the renal tissue. **(D)** Immunohistochemical staining of AGR3, CD74 and SYT11 in kidney tissues of the two groups of rats. The black arrow indicates the area of this field of view in the renal tissue. **(E)** Immunofluorescence staining of AGR3, CD74 and SYT11 in kidney tissues of the two groups of rats. The white arrow indicates the area of this field of view in the renal tissue. **(F)** Western blot detection of differences in protein expression of AGR3, CD74 and SYT11 in kidney tissues of the two groups of rats. (B; mean ± SD, n=5; F, mean ± SD, n=3; Compared with the control group: ns, *P* ≥ 0.05; *P < 0.05; **P < 0.01).

The results of animal experiments showed that the kidneys of the RF model rats were significantly swollen and had a dull color compared with the control group (**Figure 11A**). The body weight of the model group rats was slightly lower than that of the control group, but there was no statistical difference between the two groups. The SCR and BUN of the RF model rats were significantly higher than those of the control group (*P* < 0.01) (**Figure 11B**). HE and PAS staining showed renal tubule atrophy, lumen reduction, interstitial fiber hyperplasia, and a large number of inflammatory cell infiltration in model group. Masson staining showed that the collagen deposition in the kidneys of the RF model group was significantly increased (**Figure 11C**; **Supplementary Figure S2C**). The results indicated that the RF model group had significant structural and functional damage to the kidneys, as well as significant inflammatory cell infiltration and RF compared to the control group.

On this basis, we further verified the results of the bioinformatics analysis. We selected the key biomarkers of macrophage lactate metabolism and RF for verification. Immunohistochemistry and immunofluorescence showed that the expressions of AGR3, CD74 and SYT11 were significantly increased in the RF model group compared to the control group, and the areas of high expression partially overlapped with the areas of inflammatory cell infiltration (**Figures 11D, E**; **Supplementary Figures S2A, B, D–G**). Combined with the important role of macrophages in the inflammatory cell infiltration, it was further confirmed that the expressions of these key biomarkers were increased in the macrophages of the RF model kidney tissue. Finally, we detected the differences in the protein expression of AGR3, CD74 and SYT11 between the two groups by western blot. The results showed that the expressions of these key biomarkers in the kidney tissues of the RF model group were significantly higher than those of the control group (*P* < 0.05) (**Figure 11F**).

The verification results of cross-model animal experiments further verified the important role and general involvement of AGR3, CD74 and SYT11 in the progression of RF. Moreover, the results also partially demonstrated their correlation with the infiltration of inflammatory cells such as macrophages, which provided a reference and basis for improving RF from the perspective of macrophage lactate metabolism.

## 4 Discussion

CKD is a chronic progressive disease that seriously endangers human health. How to effectively delay RF is very important for the treatment of CKD. The close relationship between macrophages and lactic acid metabolism and the occurrence and development of RF provides a new idea for us to further explore the mechanism of RF and develop targeted treatment strategies. Therefore, this study found and validated new biomarkers related to RF through bioinformatics analysis and animal experiments, and explored the molecular mechanisms of these biomarkers.

This study identified three biomarkers (AGR3, CD74, and SYT11) related to macrophage and lactate metabolism for the first time. AGR3 belongs to the Anterior GRadient protein family, which includes AGR1, AGR2, and AGR3, and is mainly involved in endoplasmic reticulum secretion and the biogenesis of transmembrane proteins (48, 49). Although there are no reports on the role of AGR3 in RF yet. However, considering its role in promoting cell proliferation, which aligns with the pathological mechanism of fibrosis (50). It is also suggested that AGR3 may be a new target worth exploring in macrophage lactate metabolism and RF. CD74 is a type II transmembrane protein mainly expressed on antigen-presenting cells (APCs) such as macrophages, it can regulate the proliferation, survival, and secretion of inflammatory and fibrotic mediators in non-immune and non-tumor cells (51). The study found that the knockout of CD74 could alleviate glomerular damage induced by anti-GBM antiserum (52). However, there are two opposite results regarding its role in RF (51, 53). The role of CD74 in the activation of immune cells has been reported in systemic lupus erythematosus (54). However, this study found for the first time that it was positively correlated with activated dendritic cells in RF, suggesting that it may promote fibrosis through the antigen presentation pathway. SYT11 is a member of the synaptotagmin family, which is associated with susceptibility to Parkinson’s disease (PD) and schizophrenia (55, 56). In recent years, studies have found that SYT11 can also regulate Golgi morphology and vesicle transport, thereby altering the ECM and promoting epithelial-mesenchymal transition (EMT) (57). If SYT11 inhibits the secretion or activity of matrix metalloproteinases, it may lead to a reduction in ECM degradation and promote fibrotic deposition (58). This study reveals its strong correlation with activated CD4+ T cells, suggesting that it may be involved in the T cell-mediated inflammatory cascade reaction in RF.

The nomogram integrating three biomarkers is significantly superior to a single biomarker, and the calibration curve shows a high degree of consistency between the predicted values and the actual values. This is consistent with the strategy of multi-gene combination models improving diagnostic accuracy in previous studies (59). Currently, the diagnosis of RF still relies on renal biopsy, and serum biomarkers (such as KIM-1, NGAL) have insufficient specificity (60). This study converts biomarkers at the gene expression level into a predictive model, which has two major advantages over traditional protein biomarkers: first, gene expression changes occur earlier than protein levels, allowing earlier reflection of the pathological process; second, it directly links macrophage activation and abnormal lactate metabolism, enabling more precise localization of the pathological mechanism.

Valine, leucine, and isoleucine are collectively known as branched-chain amino acids (BCAAs), and metabolic disorders of BCAAs may exacerbate the progression of RF (61). However, their specific role remains controversial (62). Studies have shown that the concentration of BCAAs in the tissues of mice with lupus nephritis-associated RF is significantly higher than in the control group (63). However, other researchers have found that the BCAAs in urine and the leucine and isoleucine in kidney tissue from UUO rats were reduced, and that exogenous BCAAs could significantly alleviate RF in these rats (64). Oxidative phosphorylation is the core pathway of cellular energy metabolism, particularly in the polarisation process of macrophages (65). Macrophage metabolic reprogramming is currently considered to be an important mechanism of RF, mainly manifested as the transformation of macrophages from an oxidative phosphorylation metabolic phenotype (M2 type) to a glycolytic metabolic phenotype (M1 type), thereby expressing pro-inflammatory and pro-fibrotic effects (66). Lactic acid is the main metabolite of glycolysis and can be converted to pyruvate to enter the tricarboxylic acid cycle, further demonstrating the close relationship between macrophage-lactic acid metabolism-oxidative phosphorylation-RF. Therefore, we speculate that the biomarkers AGR3, SYT11, and CD74 may all be involved in the regulation of oxidative phosphorylation and promote RF by influencing the related macrophage metabolic reprogramming. Moreover, AGR3 and SYT11 may also be involved in the progression of various factor-induced RF through pathways such as BCAA.

Macrophages are the most important immune cells in normal kidney tissues and play a dominant role in various kidney injuries and RF processes (9). The mechanism by which macrophages promote RF mainly includes the secretion of pro-inflammatory and pro-fibrotic factors and macrophage-myofibroblast transition (67). Activated B cells exacerbate RF by secreting pro-inflammatory factors and activating fibrotic signaling pathways (68). T cells and B cells, together with intrinsic fibroblasts, can form tertiary lymphoid tissues, leading to uncontrolled inflammation and delayed tissue repair, thereby exacerbating RF (69, 70). Studies have shown that various T cells (such as CD4+ T cells, T helper cell) are involved in the progression of RF (71, 72). Comprehensive literature research and analysis results suggest that AGR3, CD74, and SYT11 may all aggravate RF by affecting the expression of activated B cells, and may also participate in the process of RF through their effects on these immune cells. In addition, multiple cytokines showed significant differences between RF and control samples, and were correlated with key biomarkers. Macrophages in the inflammatory microenvironment can enhance lactate metabolism through glycolysis. The accumulation of lactate not only serves as an energy substrate but also regulates the release of pro-inflammatory factors such as TNF-α and IL-1β by modulating histone deacetylases and other mechanisms, participating in the inflammatory metabolic regulatory network (73). Moreover, these pro-inflammatory factors (such as TNF-α) also directly mediate the occurrence and maintenance of fibrosis (74). The results suggest that regulating pro-inflammatory factors may be an important medium for key genes to regulate immune cells and affect the progression of RF.

There have been numerous studies on the relationship between non-coding RNA and RF. Research has found that miR-19b-3p, which is closely related to the regulation of predicted biomarkers, can downregulate the levels of α-smooth muscle actin (α-SMA), transforming growth factor β1 (TGF-β1) and fibronectin (FN) in the kidney tissue of hyperuricemic rats, thereby reducing renal interstitial fibrosis (75). Some studies have shown that the predicted lncRNA TUG1 can act as a competing endogenous RNA to bind to miR-29b-3p of the miR-29 family, thereby blocking the inhibitory effect of miR-29b-3p on ECM synthesis and exacerbating RF (76). However, other studies have found that overexpression of TUG1 overexpression can alleviate kidney injury in diabetic nephropathy mice and reduce the inflammatory response and fibrosis of high glucose-stimulated HK-2 cells through the miR-145-5p/DUSP6 axis (77). These results suggest that TUG1 may play multiple roles in RF. Based on the research results and literature analysis, we speculate that miR-19b-3p and TUG1 may play important roles in the macrophage lactate metabolism and pro-fibrotic processes through the predicted biomarkers, but further experiments are needed to confirm this.

As a neurotransmitter reuptake inhibitor, VU0240551 is mainly used in the treatment of epilepsy, anxiety disorders, neuropathic pain and other nervous system-related diseases (78–80). The bioinformatics analysis results suggest that VU0240551 may be a potential therapeutic agent for RF. Although there have been no studies or reports of a direct association between VU0240551 and RF, this may be because the link between pathological processes associated with RF and the neurotransmitter system has not been fully explored. However, with the deepening of the research on the mechanism of the disease, and the discovery of the interaction between different systems (such as the brain-kidney axis, etc.), it is possible to reveal the potential association between VU0240551 and RF in the future, so as to provide new ideas for the treatment of RF.

This study identified the biomarkers related to macrophage and lactate metabolism in RF for the first time,and the possible mechanisms along with potential targeted therapeutic drugs were also explored. In addition, the expressions of these biomarkers were verified through animal experiments. The results of this study provide a new perspective for understanding the pathogenesis of RF and valuable targets for further exploration of targeted intervention strategies. However, in this study, we did not strictly validate the causal relationship between these biomarkers and RF. Meanwhile, there was a lack of observations in terms of functional assays. In the future, we plan to conduct in-depth mechanistic and clinical studies (such as knockout/overexpression verification and related function detection) to fully analyze the specific molecular mechanisms and clinical application value of these biomarkers in RF.

## Data Availability

The original contributions presented in the study are included in the article/**Supplementary Material**. Further inquiries can be directed to the corresponding author.

## References

[1] Lohia S Vlahou A Zoidakis J. Microbiome in chronic kidney disease (CKD): an omics perspective. Toxins. (2022) 14:176. doi: 10.3390/toxins14030176, PMID: 35324673 PMC8951538

[2] Humphreys BD. Mechanisms of renal fibrosis. Annu Rev Physiol. (2018) 80:309–26. doi: 10.1146/annurev-physiol-022516-034227, PMID: 29068765

[3] Nastase MV Zeng-Brouwers J Wygrecka M Schaefer L. Targeting renal fibrosis: Mechanisms and drug delivery systems. Adv Drug Deliv Rev. (2018) 129:295–307. doi: 10.1016/j.addr.2017.12.019, PMID: 29288033

[4] Evans M Lewis RD Morgan AR Whyte MB Hanif W Bain SC. A narrative review of chronic kidney disease in clinical practice: current challenges and future perspectives. Adv Ther. (2022) 39:33–43. doi: 10.1007/s12325-021-01927-z, PMID: 34739697 PMC8569052

[5] Rayego-Mateos S Valdivielso JM. New therapeutic targets in chronic kidney disease progression and renal fibrosis. Expert Opin Ther Targets. (2020) 24:655–70. doi: 10.1080/14728222.2020.1762173, PMID: 32338087

[6] Jurisic V Terzic T Colic S Jurisic M. The concentration of TNF-alpha correlate with number of inflammatory cells and degree of vascularization in radicular cysts. Oral Dis. (2008) 14:600–5. doi: 10.1111/j.1601-0825.2007.01426.x, PMID: 18221459

[7] Atri C Guerfali FZ Laouini D. Role of human macrophage polarization in inflammation during infectious diseases. Int J Mol Sci. (2018) 19:1801. doi: 10.3390/ijms19061801, PMID: 29921749 PMC6032107

[8] Panizo S Martínez-Arias L Alonso-Montes C Cannata P Martín-Carro B Fernández-Martín JL. Fibrosis in chronic kidney disease: pathogenesis and consequences. Int J Mol Sci. (2021) 22:408. doi: 10.3390/ijms22010408, PMID: 33401711 PMC7795409

[9] Tang PM Nikolic-Paterson DJ Lan HY. Macrophages: versatile players in renal inflammation and fibrosis. Nat Rev Nephrol. (2019) 15:144–58. doi: 10.1038/s41581-019-0110-2, PMID: 30692665

[10] Huen SC Cantley LG. Macrophages in renal injury and repair. Annu Rev Physiol. (2017) 79:449–69. doi: 10.1146/annurev-physiol-022516-034219, PMID: 28192060

[11] Peng Y Li L Shang J Zhu H Liao J Hong X. Macrophage promotes fibroblast activation and kidney fibrosis by assembling a vitronectin-enriched microenvironment. Theranostics. (2023) 13:3897–913. doi: 10.7150/thno.85250, PMID: 37441594 PMC10334827

[12] Zhang YL Tang TT Wang B Wen Y Feng Y Yin Q. Identification of a novel ECM remodeling macrophage subset in AKI to CKD transition by integrative spatial and single-cell analysis. Adv Sci (Weinheim Baden-Wurttemberg Germany). (2024) 11:e2309752. doi: 10.1002/advs.202309752, PMID: 39119903 PMC11481374

[13] Jurisic V Radenkovic S Konjevic G. The actual role of LDH as tumor marker, biochemical and clinical aspects. Adv Exp Med Biol. (2015) 867:115–24. doi: 10.1007/978-94-017-7215-0_8, PMID: 26530363

[14] Rabinowitz JD Enerbäck S. Lactate: the ugly duckling of energy metabolism. Nat Metab. (2020) 2:566–71. doi: 10.1038/s42255-020-0243-4, PMID: 32694798 PMC7983055

[15] Wu D Zhang K Khan FA Wu Q Pandupuspitasari NS Tang Y. The emerging era of lactate: A rising star in cellular signaling and its regulatory mechanisms. J Cell Biochem. (2023) 124:1067–81. doi: 10.1002/jcb.30458, PMID: 37566665

[16] Hu D Wang L Zhang Y Liu X Lu Z Li H. Sanqi oral solution ameliorates renal fibrosis by suppressing fibroblast activation via HIF-1α/PKM2/glycolysis pathway in chronic kidney disease. J Ethnopharmacol. (2024) 335:118679. doi: 10.1016/j.jep.2024.118679, PMID: 39121930

[17] Guo C Cui Y Jiao M Yao J Zhao J Tian Y. Crosstalk between proximal tubular epithelial cells and other interstitial cells in tubulointerstitial fibrosis after renal injury. Front Endocrinol. (2024) 14:1256375. doi: 10.3389/fendo.2023.1256375, PMID: 38260142 PMC10801024

[18] Xu B Liu Y Li N Geng Q. Lactate and lactylation in macrophage metabolic reprogramming: current progress and outstanding issues. Front Immunol. (2024) 15:1395786. doi: 10.3389/fimmu.2024.1395786, PMID: 38835758 PMC11148263

[19] Zhou HC Yu WW Yan XY Liang XQ Ma XF Long JP. Lactate-driven macrophage polarization in the inflammatory microenvironment alleviates intestinal inflammation. Front Immunol. (2022) 13:1013686. doi: 10.3389/fimmu.2022.1013686, PMID: 36330516 PMC9623299

[20] Davis S Meltzer PS. GEOquery: a bridge between the gene expression omnibus (GEO) and BioConductor. Bioinf (Oxford England). (2007) 23:1846–7. doi: 10.1093/bioinformatics/btm254, PMID: 17496320

[21] Hänzelmann S Castelo R Guinney J. GSVA: gene set variation analysis for microarray and RNA-seq data. BMC Bioinf. (2013) 14:7. doi: 10.1186/1471-2105-14-7, PMID: 23323831 PMC3618321

[22] Charoentong P Finotello F Angelova M Mayer C Efremova M Rieder D. Pan-cancer immunogenomic analyses reveal genotype-immunophenotype relationships and predictors of response to checkpoint blockade. Cell Rep. (2017) 18:248–62. doi: 10.1016/j.celrep.2016.12.019, PMID: 28052254

[23] Langfelder P Horvath S. WGCNA: an R package for weighted correlation network analysis. BMC Bioinf. (2008) 9:559. doi: 10.1186/1471-2105-9-559, PMID: 19114008 PMC2631488

[24] Ritchie ME Phipson B Wu D Hu Y Law CW Shi W. limma powers differential expression analyses for RNA-sequencing and microarray studies. Nucleic Acids Res. (2015) 43:e47. doi: 10.1093/nar/gkv007, PMID: 25605792 PMC4402510

[25] Gustavsson EK Zhang D Reynolds RH Garcia-Ruiz S Ryten M. ggtranscript: an R package for the visualization and interpretation of transcript isoforms using ggplot2. Bioinf (Oxford England). (2022) 38:3844–6. doi: 10.1101/2022.03.28.486050, PMID: 35751589 PMC9344834

[26] Gu Z Hübschmann D. Make interactive complex heatmaps in R. Bioinf (Oxford England). (2022) 38:1460–2. doi: 10.1093/bioinformatics/btab806, PMID: 34864868 PMC8826183

[27] Zheng Y Gao W Zhang Q Cheng X Liu Y Qi Z. Ferroptosis and autophagy-related genes in the pathogenesis of ischemic cardiomyopathy. Front Cardiovasc Med. (2022) 9:906753. doi: 10.3389/fcvm.2022.906753, PMID: 35845045 PMC9279674

[28] Yu G Wang LG Han Y He QY. clusterProfiler: an R package for comparing biological themes among gene clusters. Omics: J Integr Biol. (2012) 16:284–7. doi: 10.1089/omi.2011.0118, PMID: 22455463 PMC3339379

[29] Shannon P Markiel A Ozier O Baliga NS Wang JT Ramage D. Cytoscape: a software environment for integrated models of biomolecular interaction networks. Genome Res. (2003) 13:2498–504. doi: 10.1101/gr.1239303, PMID: 14597658 PMC403769

[30] Friedman J Hastie T Tibshirani R. Regularization paths for generalized linear models via coordinate descent. J Stat Softw. (2010) 33:1–22. doi: 10.18637/jss.v033.i01, PMID: 20808728 PMC2929880

[31] Wang X Jiang G Zong J Lv D Lu M Qu X. Revealing the novel ferroptosis-related therapeutic targets for diabetic foot ulcer based on the machine learning. Front Genet. (2022) 13:944425. doi: 10.3389/fgene.2022.944425, PMID: 36226171 PMC9549267

[32] Robin X Turck N Hainard A Tiberti N Lisacek F Sanchez JC. pROC: an open-source package for R and S+ to analyze and compare ROC curves. BMC Bioinf. (2011) 12:77. doi: 10.1186/1471-2105-12-77, PMID: 21414208 PMC3068975

[33] Ma X Cheng J Zhao P Li L Tao K Chen H. DNA methylation profiling to predict recurrence risk in stage I lung adenocarcinoma: Development and validation of a nomogram to clinical management. J Cell Mol Med. (2020) 24:7576–89. doi: 10.1111/jcmm.15393, PMID: 32530136 PMC7339160

[34] Zhu H Hu H Hao B Zhan W Yan T Zhang J. Insights into a machine learning-based palmitoylation-related gene model for predicting the prognosis and treatment response of breast cancer patients. Technol Cancer Res Treat. (2024) 23:15330338241263434. doi: 10.1177/15330338241263434, PMID: 39205467 PMC11363247

[35] Nazari-Ghadikolaei A Fikse F Gelinder Viklund Å Eriksson S. Factor analysis of evaluated and linearly scored traits in Swedish Warmblood horses. J Anim Breed Genet = Z fur Tierzuchtung und Zuchtungsbiologie. (2023) 140:366–75. doi: 10.1111/jbg.12764, PMID: 36852464

[36] Koleva-Georgieva DN Sivkova NP Terzieva D. Serum inflammatory cytokines IL-1beta, IL-6, TNF-alpha and VEGF have influence on the development of diabetic retinopathy. Folia Med. (2011) 53:44–50. doi: 10.2478/v10153-010-0036-8, PMID: 21797106

[37] Yang L He T Yu Y. Uric acid promotes interleukin-17 expression to cause kidney injury. J Biochem Mol Toxicol. (2024) 38:e23550. doi: 10.1002/jbt.23550, PMID: 37815028

[38] Bermejo-Martin JF García-Mateo N Motos A Resino S Tamayo L Ryan Murua P. Effect of viral storm in patients admitted to intensive care units with severe COVID-19 in Spain: a multicentre, prospective, cohort study. Lancet Microbe. (2023) 4:e431–41. doi: 10.1016/S2666-5247(23)00041-1, PMID: 37116517 PMC10129133

[39] Liang X Peng Z Deng Y Lin X Chen R Niu Y. The role of T cells and shared genes in psoriasis and inflammatory bowel disease based on single-cell RNA and comprehensive analysis. Immunobiology. (2023) 228:152754. doi: 10.1016/j.imbio.2023.152754, PMID: 37806279

[40] Gao J Deng Q Yu J Wang C Wei W. Role of renal tubular epithelial cells and macrophages in cisplatin-induced acute renal injury. Life Sci. (2024) 339:122450. doi: 10.1016/j.lfs.2024.122450, PMID: 38262575

[41] Zhang C Sun C Zhao Y Ye B Yu G. Signaling pathways of liver regeneration: Biological mechanisms and implications. iScience. (2023) 27:108683. doi: 10.1016/j.isci.2023.108683, PMID: 38155779 PMC10753089

[42] Wilkerson MD Hayes DN. ConsensusClusterPlus: a class discovery tool with confidence assessments and item tracking. Bioinf (Oxford England). (2010) 26:1572–3. doi: 10.1093/bioinformatics/btq170, PMID: 20427518 PMC2881355

[43] Yong C Huang G Ge H Zhu Y Yang Y Yu Y. Perilla frutescens L. alleviates trimethylamine N-oxide-induced apoptosis in the renal tubule by regulating ASK1-JNK phosphorylation. Phytother Res: PTR. (2023) 37:1274–92. doi: 10.1002/ptr.7684, PMID: 36420586

[44] Ge H Wei Y Zhang W Yong C Chen Y Zhou E. Suyin Detoxification Granule alleviates trimethylamine N-oxide-induced tubular ferroptosis and renal fibrosis to prevent chronic kidney disease progression. Phytomed: Int J Phytother Phytopharmacol. (2024) 135:156195. doi: 10.1016/j.phymed.2024.156195, PMID: 39488871

[45] Shu L Quan L Wang Y Chen Y Yong C Tian F. Suyin detoxification prescription regulates the Klotho and ERK/NF-κB signaling pathways to alleviate renal injury. Cell Biochem Biophys. (2025). doi: 10.1007/s12013-025-01695-5. Advance online publication., PMID: 39966333

[46] Tanaskovic I Ilic S Jurisic V Lackovic M Milosavljevic Z Stankovic V. Histochemical, immunohistochemical and ultrastructural analysis of aortic wall in neonatal coarctation. Romanian J Morphol Embryol = Rev Roumaine Morphol Embryol. (2019) 60:1291–8., PMID: 32239107

[47] Jurisic V Srdic-Rajic T Konjevic G Bogdanovic G Colic M. TNF-α induced apoptosis is accompanied with rapid CD30 and slower CD45 shedding from K-562 cells. J Membr Biol. (2011) 239:115–22. doi: 10.1007/s00232-010-9309-7, PMID: 21221555

[48] Boisteau E Posseme C Di Modugno F Edeline J Coulouarn C Hrstka R. Anterior gradient proteins in gastrointestinal cancers: from cell biology to pathophysiology. Oncogene. (2022) 41:4673–85. doi: 10.1038/s41388-022-02452-1, PMID: 36068336

[49] Ye R Wang C Sun P Bai S Zhao L. AGR3 regulates airway epithelial junctions in patients with frequent exacerbations of COPD. Front Pharmacol. (2021) 12:669403. doi: 10.3389/fphar.2021.669403, PMID: 34177583 PMC8232749

[50] Jian L Xie J Guo S Yu H Chen R Tao K. AGR3 promotes estrogen receptor-positive breast cancer cell proliferation in an estrogen-dependent manner. Oncol Lett. (2020) 20:1441–51. doi: 10.3892/ol.2020.11683, PMID: 32724387 PMC7377037

[51] Zhou JX Cheng AS Chen L Li LX Agborbesong E Torres VE. CD74 promotes cyst growth and renal fibrosis in autosomal dominant polycystic kidney disease. Cells. (2024) 13:489. doi: 10.3390/cells13060489, PMID: 38534333 PMC10968819

[52] Djudjaj S Lue H Rong S Papasotiriou M Klinkhammer BM Zok S. Macrophage migration inhibitory factor mediates proliferative GN via CD74. J Am Soc Nephrol: JASN. (2016) 27:1650–64. doi: 10.1681/ASN.2015020149, PMID: 26453615 PMC4884103

[53] Valiño-Rivas L Baeza-Bermejillo C Gonzalez-Lafuente L Sanz AB Ortiz A Sanchez-Niño MD. CD74 in kidney disease. Front Immunol. (2015) 6:483. doi: 10.3389/fimmu.2015.00483, PMID: 26441987 PMC4585214

[54] Tselios K Wakani L Gladman DD Su J Urowitz MB. Response to placebo in non-renal, non-neuropsychiatric systemic lupus erythematosus: a systematic review and pooled analysis. Rheumatol (Oxford England). (2021) 60:73–80. doi: 10.1093/rheumatology/keaa655, PMID: 33140092

[55] Sesar A Cacheiro P López-López M Camiña-Tato M Quintáns B Monroy-Jaramillo N. Synaptotagmin XI in Parkinson’s disease: New evidence from an association study in Spain and Mexico. J Neurol Sci. (2016) 362:321–5. doi: 10.1016/j.jns.2016.02.014, PMID: 26944171

[56] Inoue S Imamura A Okazaki Y Yokota H Arai M Hayashi N. Synaptotagmin XI as a candidate gene for susceptibility to schizophrenia. Am J Med Genet Part B Neuropsychiatr Genet: Off Publ Int Soc Psychiatr Genet. (2007) 144B:332–40. doi: 10.1002/ajmg.b.30465, PMID: 17192956

[57] Bajaj R Rodriguez BL Russell WK Warner AN Diao L Wang J. Impad1 and Syt11 work in an epistatic pathway that regulates EMT-mediated vesicular trafficking to drive lung cancer invasion and metastasis. Cell Rep. (2022) 40:111429. doi: 10.1016/j.celrep.2022.111429, PMID: 36170810 PMC9665355

[58] Scott LE Weinberg SH Lemmon CA. Mechanochemical signaling of the extracellular matrix in epithelial-mesenchymal transition. Front Cell Dev Biol. (2019) 7:135. doi: 10.3389/fcell.2019.00135, PMID: 31380370 PMC6658819

[59] Huang G Xu X Ju C Zhong N He J Tang XX. Identification and validation of autophagy-related gene expression for predicting prognosis in patients with idiopathic pulmonary fibrosis. Front Immunol. (2022) 13:997138. doi: 10.3389/fimmu.2022.997138, PMID: 36211385 PMC9533718

[60] Li L Fu H Liu Y. The fibrogenic niche in kidney fibrosis: components and mechanisms. Nat Rev Nephrol. (2022) 18:545–57. doi: 10.1038/s41581-022-00590-z, PMID: 35788561

[61] Zhang C Huang H Li C Wei L Wu J Wang R. Transcriptomics and UHPLC-QQQ-MS analyses reveal the dysregulation of branched chain amino acids metabolism in renal fibrotic rats. J Pharm Biomed Anal. (2024) 245:116197. doi: 10.1016/j.jpba.2024.116197, PMID: 38723558

[62] Wu T Wang M Ning F Zhou S Hu X Xin H. Emerging role for branched-chain amino acids metabolism in fibrosis. Pharmacol Res. (2023) 187:106604. doi: 10.1016/j.phrs.2022.106604, PMID: 36503000

[63] Alexander JJ Zwingmann C Jacob A Quigg R. Alteration in kidney glucose and amino acids are implicated in renal pathology in MRL/lpr mice. Biochim Biophys Acta. (2007) 1772:1143–9. doi: 10.1016/j.bbadis.2007.07.002, PMID: 17942282

[64] Zhao L Dong M Liao S Du Y Zhou Q Zheng H. Identification of key metabolic changes in renal interstitial fibrosis rats using metabonomics and pharmacology. Sci Rep. (2016) 6:27194. doi: 10.1038/srep27194, PMID: 27256510 PMC4891668

[65] Viola A Munari F Sánchez-Rodríguez R Scolaro T Castegna A. The metabolic signature of macrophage responses. Front Immunol. (2019) 10:1462. doi: 10.3389/fimmu.2019.01462, PMID: 31333642 PMC6618143

[66] Wang S Liu G Li Y Pan Y. Metabolic reprogramming induces macrophage polarization in the tumor microenvironment. Front Immunol. (2022) 13:840029. doi: 10.3389/fimmu.2022.840029, PMID: 35874739 PMC9302576

[67] Wei J Xu Z Yan X. The role of the macrophage-to-myofibroblast transition in renal fibrosis. Front Immunol. (2022) 13:934377. doi: 10.3389/fimmu.2022.934377, PMID: 35990655 PMC9389037

[68] Lin Y Cai F Wang X Yang Y Ren Y Yao C. FADD phosphorylation contributes to development of renal fibrosis by accelerating epithelial-mesenchymal transition. Cell Cycle (Georgetown Tex). (2023) 22:580–95. doi: 10.1080/15384101.2022.2136463, PMID: 36281535 PMC9928456

[69] Sato Y Silina K van den Broek M Hirahara K Yanagita M. The roles of tertiary lymphoid structures in chronic diseases. Nat Rev Nephrol. (2023) 19:525–37. doi: 10.1038/s41581-023-00706-z, PMID: 37046081 PMC10092939

[70] Luo R Chang D Zhang N Cheng Y Ge S Xu G. T follicular helper cells in tertiary lymphoid structure contribute to renal fibrosis by IL-21. Int J Mol Sci. (2023) 24:12535. doi: 10.3390/ijms241612535, PMID: 37628716 PMC10454845

[71] Paust HJ Song N De Feo D Asada N Tuzlak S Zhao Y. CD4+ T cells produce GM-CSF and drive immune-mediated glomerular disease by licensing monocyte-derived cells to produce MMP12. Sci Trans Med. (2023) 15:eadd6137. doi: 10.1126/scitranslmed.add6137, PMID: 36921033

[72] Zhang M Zhang S. T cells in fibrosis and fibrotic diseases. Front Immunol. (2020) 11:1142. doi: 10.3389/fimmu.2020.01142, PMID: 32676074 PMC7333347

[73] Jurisic V Bumbasirevic V Konjevic G Djuricic B Spuzic I. TNF-alpha induces changes in LDH isotype profile following triggering of apoptosis in PBL of non-Hodgkin’s lymphomas. Ann Hematol. (2004) 83:84–91. doi: 10.1007/s00277-003-0731-0, PMID: 14586559

[74] Jurisic V Terzic T Pavlovic S Colovic N Colovic M. Elevated TNF-alpha and LDH without parathormone disturbance is associated with diffuse osteolytic lesions in leukemic transformation of myelofibrosis. Pathol Res Pract. (2008) 204:129–32. doi: 10.1016/j.prp.2007.09.001, PMID: 17976926

[75] Hu L Yang K Mai X Wei J Ma C. Depleted HDAC3 attenuates hyperuricemia-induced renal interstitial fibrosis via miR-19b-3p/SF3B3 axis. Cell Cycle (Georgetown Tex). (2022) 21:450–61. doi: 10.1080/15384101.2021.1989899, PMID: 35025700 PMC8942505

[76] Zhang J Zhang Y Gao J Wang M Li X Cui Z. Long noncoding RNA tug1 promotes angiotensin II-induced renal fibrosis by binding to mineralocorticoid receptor and negatively regulating MicroR-29b-3p. Hypertens (Dallas Tex: 1979). (2021) 78:693–705. doi: 10.1161/HYPERTENSIONAHA.120.16395, PMID: 34333990

[77] Wang T Cui S Liu X Han L Duan X Feng S. LncTUG1 ameliorates renal tubular fibrosis in experimental diabetic nephropathy through the miR-145-5p/dual-specificity phosphatase 6 axis. Renal Fail. (2023) 45:2173950. doi: 10.1080/0886022X.2023.2173950, PMID: 36794657 PMC9937007

[78] Ford A Castonguay A Cottet M Little JW Chen Z Symons-Liguori AM. Engagement of the GABA to KCC2 signaling pathway contributes to the analgesic effects of A3AR agonists in neuropathic pain. J Neurosci: Off J Soc Neurosci. (2015) 35:6057–67. doi: 10.1523/JNEUROSCI.4495-14.2015, PMID: 25878279 PMC4397603

[79] Hamidi S Avoli M. KCC2 function modulates *in vitro* ictogenesis. Neurobiol Dis. (2015) 79:51–8. doi: 10.1016/j.nbd.2015.04.006, PMID: 25926348 PMC4880462

[80] Zhang H Xu L Xiong J Li X Yang Y Liu Y. Role of KCC2 in the regulation of brain-derived neurotrophic factor on ethanol consumption in rats. Mol Neurobiol. (2023) 60:1040–9. doi: 10.1007/s12035-022-03126-5, PMID: 36401060

