# Supplementary figures and images for: To reveal biomarkers related to macrophage and lactic acid metabolism in renal fibrosis and explore their mechanisms
Source: Front Immunol. 2025 Jul 18;16:1609903. doi: 10.3389/fimmu.2025.1609903 (PMC12313614; doi:10.3389/fimmu.2025.1609903)

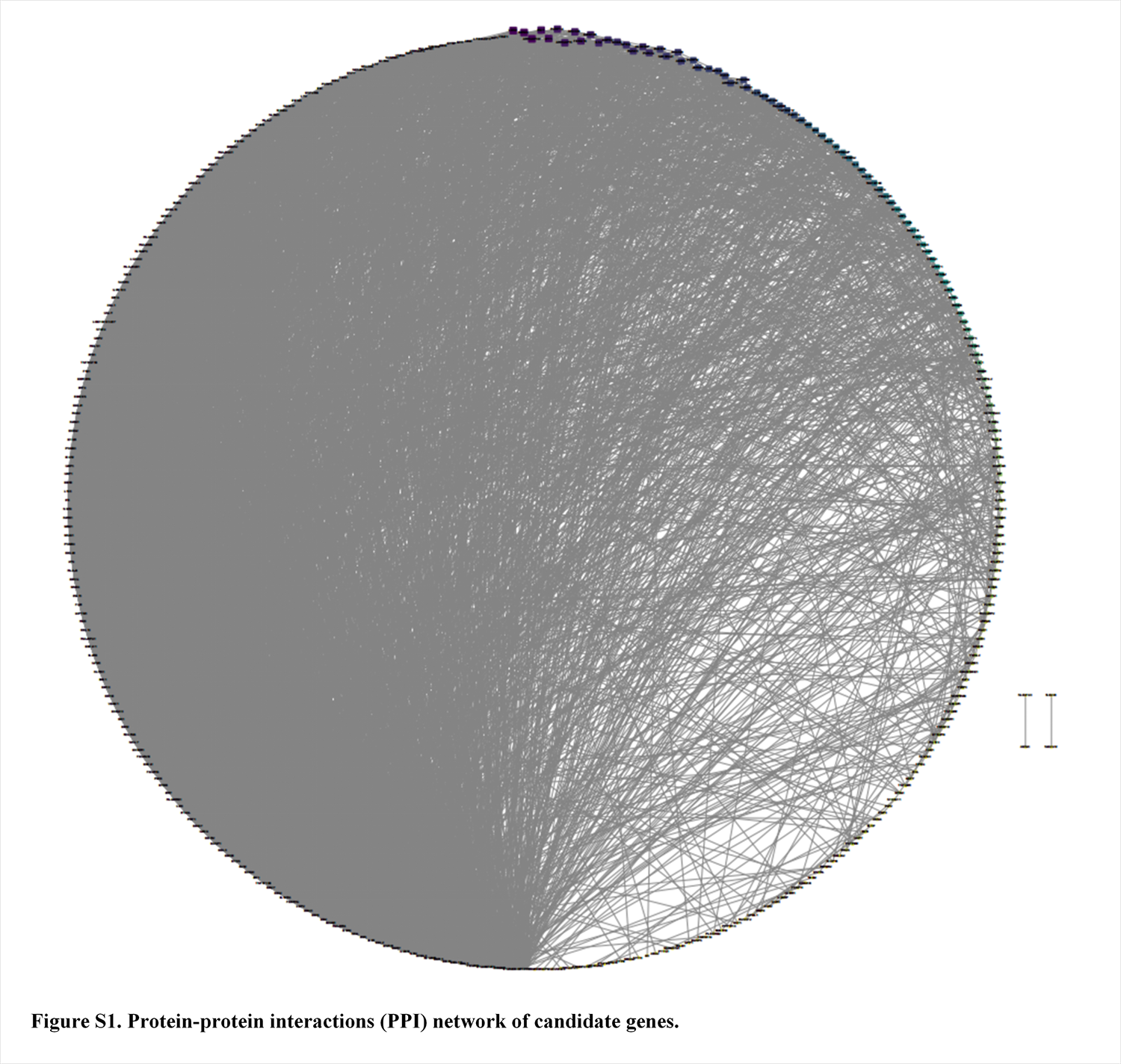

Supplement: Supplementary file 5 [file Image1.tif]

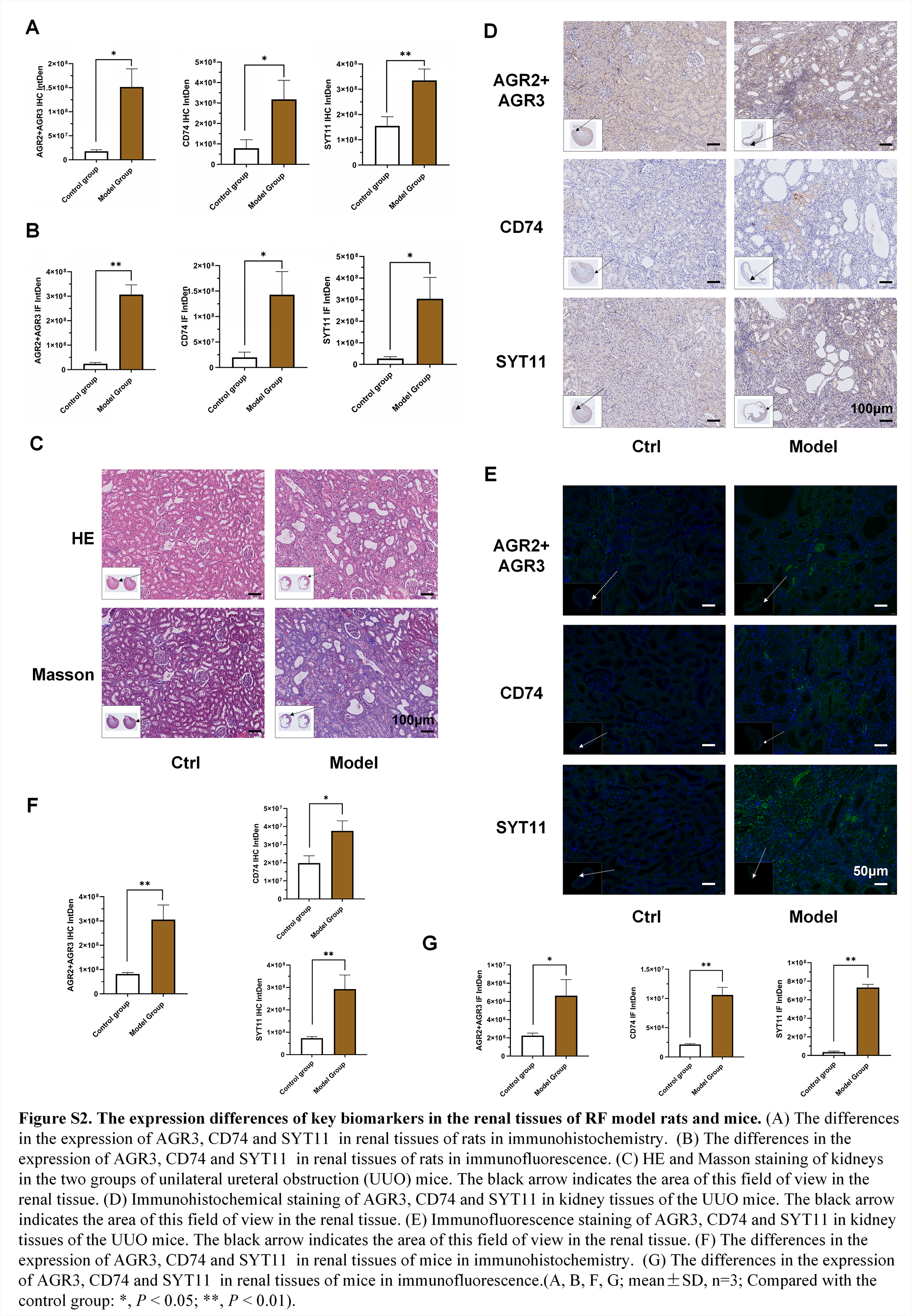

Supplement: Supplementary file 6 [file Image2.tif]
